# Radioresistant intrathymic stem cells: retrospective analysis and concept of the role in thymic oncogenesis and post-irradiation regeneration

**DOI:** 10.3389/fimmu.2026.1748485

**Published:** 2026-02-25

**Authors:** Valentin P. Shichkin

**Affiliations:** Aktipharm LLC, Kyiv, Ukraine

**Keywords:** cytokines, dormant stem cells, intrathymic stem cells, radio resistance, thymic oncogenesis, thymocyte growth factor, thymus post-radiation regeneration, T-lymphocyte precursors

## Abstract

Radioresistant thymic cells encompass minor subsets of lymphoid precursors of T cells (TLPs), innate lymphoid cells (ILCs), as well as stromal-epithelial and endothelial populations. This review focuses on radioresistant TLPs and their regenerative and functional roles in thymic regeneration following damaging influences, particularly irradiation, as well as their secretory product, referred to as thymocyte growth factor (THGF). Retrospective analysis of experimental data assumes that THGF-producing and THGF-responsive cells correspond to the earliest stage of thymocyte precursors, double-negative (DN) TLPs, of CD117^-^Thy-1^+^Sca-1^+^CD44^+^CD25^-^CD4^-^CD8^-^ phenotype, and may be a target for thymic oncogenesis, when they are in the activated DN1→DN2 stage. Unique features of THGF-driven proliferation of these cells include a colchicine-resistant DNA synthesis and, presumably, the formation of a “daughter” cell pool within “mother” cell-like structures, as well as the formation of colony-cluster-like structures, which are presumably composed mainly of single activated mother DN1 and surrounding daughter TLPs progressing from DN2 to DN4 stage. This atypical proliferation mode may represent an evolutionarily conserved mechanism of “defended mitosis” and/or amitotic or endomitotic pathways division, protecting against radiation-induced injury and thus allowing the cell expansion. THGF, which is induced by γ-irradiation and appears essential for autocrine expansion of radioresistant TLPs, initiates a cascade that enables subsequent responsiveness to IL-7, SCF, IL-2, and additional cytokines. The presented analysis proposes the concept of intrathymic dormant stem cells, which become activated under extreme conditions, and insights into parallels between THGF-responsive cells and other radioresistant thymic populations, suggesting an integrated network of stromal and lymphoid elements that orchestrate thymic regeneration. Together, this review proposes a model in which THGF acts as a critical regulator of dormant intrathymic stem cells, enabling their activation, protected proliferation, and differentiation, and thereby contributing crucially to the lymphoid lineage of thymic regeneration after irradiation, in addition to the concept of the IL-22-dependent pathway of stromal-epithelial regeneration of intrathymic niches microenvironment.

## 1 Introduction

The thymus is a central organ responsible for T-cell differentiation, maturation, and both positive and negative selection. These processes ensure the generation of functionally competent and self-tolerant T lymphocytes, which play a pivotal role in cellular immunity, including the recognition of tumor cells, foreign antigens, and pathogens. Recent studies have expanded the thymus’s role beyond T-cell development, suggesting its contribution to memory B-cell differentiation through an unconventional pathway independent of external antigen exposure (1). Together with medullary thymic epithelial cells (TECs), dendritic cells, and macrophages, these B cells support negative T-cell selection, immune homeostasis, and the regulation of autoimmunity, particularly during aging (2).

The thymus is highly sensitive to various forms of stress, both via neural regulation (3) and direct physical or chemical injury (4–6). Such insults result in profound structural and functional impairment, disrupting normal T-cell development and leading to increased susceptibility to cancer, autoimmune, allergic, and infectious diseases, as well as accelerated immunosenescence. Nevertheless, the thymus possesses a remarkable capacity for self-renewal due to the presence of thymic stem cells (TSCs), which are key to structural regeneration and functional recovery under both physiological and stress-induced conditions (7, 8).

However, despite the long history, the mechanisms contributing to endogenous thymic regeneration were not well understood. Recent studies on mouse models have reported multiple pathways of thymic regeneration and the molecular mechanisms that trigger these pathways following various damaging treatments (9). These investigations have demonstrated also the important role of regulatory T (Treg)-cell balance in the thymic recovery via expression of various regenerative factors, in particular, the cytokine amphiregulin (10). Furthermore, an analogous population of Treg cells (CD39^+^ICOS^+^) was identified by these authors also in the human thymus, inciting their new function and potential in therapeutic applications associated with aging- and treatment-induced immunosuppression.

T-cell development occurs within specialized thymic microenvironments known as niches, where immature thymocytes interact with TECs and other stromal elements. These interactions guide thymocyte differentiation through distinct developmental stages, ensuring the generation of mature, functional, and self-tolerant T cells (11–13). However, age-related epithelial defects limit thymic function (14, 15) and impair regeneration following injury (16).

While current research has primarily focused on TECs and thymic epithelial stem cells (TESCs) as the principal components responsible for thymic renewal (12, 16–26), intrathymic T-lymphocyte precursors (TLPs) exhibiting stem-like properties have received comparatively little attention in the context of thymic recovery. Some investigators have proposed that the bone marrow contains a population of universal dormant hematopoietic stem cells (HSCs), which can be activated under severe stress and serve as progenitors not only for TLPs but also for epithelial and stromal compartments, depending on local epigenetic cues (27–29). This hypothesis is supported by several experimental evidence showing the generation of epithelial tissues in the lungs, liver, and intestine from transplanted highly purified bone marrow or cord blood HSCs (29–34). Although intriguing, this concept requires further verification, as most researchers support the view that discrete stem cell populations exist for lymphoid and stromal-epithelial lineages.

Thymocyte development begins with early CD25^-^CD44^+^ TLPs, also referred to as CD4^-^CD8^-^ double-negative 1 (DN1) cells, which originate from multipotent HSCs migrating from embryonic sources such as the aorta–gonad–mesonephros region, yolk sac, and fetal liver, and later from the adult bone marrow. Intrathymic TLPs are localized mainly in the subcapsular cortical zone, although some studies also indicate their presence near the paracortical region (8, 13).

Entry of these progenitors into the thymus requires expression of CCR7 and CCR9 and responsiveness to Notch signaling (13, 35, 36). Early T-cell maturation occurs primarily in the thymic cortex through interactions with cortical TECs, which produce chemokines CCL25 and CXCL12, cytokines interleukin-7 (IL-7) and stem cell factor (SCF), and the Notch ligand Dll4 - all essential for thymocyte survival and differentiation (7, 20, 37, 38). At the DN1-DN2 stages, survival is regulated by IL-7 and SCF through their receptors IL-7R and c-kit (CD117), respectively, activating anti-apoptotic Bcl-2 signaling (13, 39). DN2 TLPs (CD4^-^CD8^-^CD25^+^CD44^+^) proliferate, downregulate CD44, and transition into DN3 cells (CD4^-^CD8^-^CD25^+^CD44^-^), where TCRβ rearrangement occurs and B-cell potential is lost (40, 41). Subsequent DN4 cells (CD4^-^CD8^-^CD25^-^CD44^-^) proliferate in the subcapsular zone, migrate to the cortex, and differentiate into double-positive (DP) CD4^+^CD8^+^ thymocytes (13, 42), which express the mature TCRαβ/CD3 complex and undergo negative selection. DP thymocytes then move into the medulla, where positive selection yields single-positive (SP) naïve T cells that subsequently migrate to the periphery (8, 40, 41).

Both αβ and γδ T-cell lineages arise in the thymus from common uncommitted early T-cell precursors (ETPs), derived from bone marrow HSCs and represented by DN1 TLPs (CD44^+^CD25^-^CD4^-^CD8^-^B220^-^CD11b^-^CD11c^-^NK1.1^-^TCRβ^-^TCRγδ^-^), including both CD117^+^ and CD117^-/lo^ subpopulations (43). Among these, only the CD117^+^ DN1 fraction represents true TLPs capable of progressing to DN2 and DN3 stages and generating DP αβ thymocytes (42). These cells retain limited NK potential (44) and express transcriptional regulators associated with stemness and early T-cell identity (45). Divergence of αβ and γδ T-cell lineages occurs at the DN3 stage (43).

Based on CD24 expression, CD117^+^ DN1 ETPs are subdivided into CD24^-^ (DN1a) and CD24^lo^ (DN1b) subsets, thought to have a precursor–progeny relationship (44). Within the ETP pool, a CD63^+^Ly6c^+^ subpopulation has been identified as a granulocyte-committed precursor lacking T-cell potential (43). The γδ lineage is believed to diverge from the main developmental pathway at the DN2–DN3 transition, when TCRβ/γ/δ gene rearrangement occurs; this bipotency is lost by DN3 (46). The current model of early T-cell development posits that ETPs (DN1 thymocytes) represent the most immature thymic population, progressing to DN2 where lineage commitment toward αβ or γδ T cells is initiated, and finalized at DN3 during β-selection (expression of functional TCRβ/pre-Tα complex) or γδ TCR dimer formation (43).

Recent studies show that some γδ T cells may also arise from CD117^-^ DN1 thymocytes. Single-cell transcriptomic analyses revealed multiple DN1 subpopulations with preferential differentiation toward IL-17- or IFNγ-producing γδ T cells (43). These CD117^-^ DN1 cells can be further subdivided into CD117^lo^CD24^hi^ (DN1c), CD117^-^CD24^hi^ (DN1d), and CD117^-^CD24^-^ (DN1e) subsets (43), previously not considered part of the canonical T-cell developmental pathway. However, it was demonstrated that IL-17-producing γδ T cells derive from Sox13^+^ DN1d thymocytes rather than from ETPs, bypassing the classic ETP–DN2–DN3 sequence (47). Furthermore, TCR signal strength influences γδ T-cell fate: weak signaling promotes IL-17A, whereas strong signaling induces an IFNγ phenotype (48).

A particularly intriguing and underexplored aspect of thymic biology is the existence of radioresistant stem cells found among both TLP and TESC populations (49–64). These radioresistant TSCs can survive and maintain their regenerative capacity even after exposure to lethal irradiation, demonstrating self-renewal, plasticity, and multilineage differentiation potential (7, 49, 53–55, 57), highlighting the critical role of radioresistant TSCs in the early autonomous restoration of thymic architecture following irradiation damage.

Modern studies, focusing on the radioresistance mechanisms of thymic stromal cells and TESCs, demonstrated the activation of DNA repair mechanisms, antioxidant defenses, and stress-response signaling pathways (9, 56–63). It was reported that proteins such as p53 and ATM, as well as antioxidant enzymes like superoxide dismutase, play key roles in cellular protection (56, 59). Additionally, the activation of survival pathways PI3K/AKT and MAPK, along with stress-response regulators ATF4, promotes resistance to radiation-induced apoptosis (60).

As early as the 1960s, it was recognized that certain thymic TLPs exhibit substantial radioresistance (65). In 1975, Kadish and Basch reported the existence of a local population of radioresistant intrathymic TLPs capable of driving post-irradiation thymic regeneration independently of precursors migrated from bone marrow (49). These radioresistant TLPs represented a minor subset of CD4^-^CD8^-^ DN intrathymic TLPs (early referred to as the L3T4^-^Lyt2^-^ TLPs), located primarily in the subcapsular zone of the thymic cortex and capable of differentiating into both CD4^+^ and CD8^+^ T cells (4, 49, 52, 54, 55, 66–70).

While radioresistant intrathymic TLPs have now largely remained outside researchers’ attention in contrast to the stromal-epithelial thymic compartment, two fundamental properties of these TLPs, stem cell-like potential and functional resilience under damaging conditions, underscore their biological significance within the thymic microenvironment. These TLPs contribute to thymic regeneration through cytokine secretion and intercellular interactions, impacting the stromal-epithelial compartment, as well as through their differentiation into mature thymocytes (8, 37, 52, 71–73).

In 1983, we established two transformed thymic cell lines, TC-SC-1/1.1 and TC.SC-1/2.0, with a phenotype characteristic of intrathymic TLPs, expressing stem cell 1 antigen (SC-1) (74). These cell lines, under stimulation with gamma-irradiation, produced an unknown growth activity, identified by us in 1984 and later named a thymocyte growth factor (THGF) (50, 53, 72, 75, 76). These cell lines, as well as their secretory product, THGF, and its target cells were intensively explored from 1983 to 1991, and several additional important data were obtained later, in 1999. The results of these research findings were presented in detail in four doctoral theses, and the main data were published in scientific journals, primarily in Russian-language journals, and remain poorly accessible to broad scientific discussion. TC-SC-1/1.1 and TC.SC-1/2.0 cell lines were registered and stored in the Official Cell Line Collection at the Institute of Cytology, Russian Academy of Sciences (St. Petersburg, Russia) since 1987.

This article aims to conduct a retrospective analysis of our findings and reinterpret them through the lens of modern knowledge in immunology and stem cell biology in the context of the presumable role of THGF activity and its comparison with other cytokines. The updated publication of the combined data may motivate further research, also by independent research groups, which were interrupted due to a range of critical circumstances, and lead to a deeper understanding of TLP radioresistance mechanisms and their functional roles in the endogenous thymic regeneration after damage treatments, especially irradiation injury. In turn, this could open new avenues for therapeutic interventions and radioprotection in the context of thymic dysfunction and thymic-associated immunosenescence. The discussed experimental data are related mainly to the TC.SC-1/2.0 cell line, which was used as the basic experimental model of intrathymic TLPs.

## 2 Stem cell identification markers: from history to modern

Historically, the SC-1 antigen, initially identified using rabbit antisera against mouse brain, was used as a marker of HSCs and intrathymic TLPs (77–79). In some TLP populations, it was co-expressed with the Thy-1 antigen (80–82). Other TLP identifying markers included glycan receptors for the galactose-specific lectin peanut agglutinin (PNA-R) (67, 83, 84) and interleukin-2 (IL-2) (85), along with the absence of mature T-cell markers L3T4 (CD4) and Lyt-2 (CD8) (37, 86) (**Table 1**).

**Table 1:** Modern cell markers and their historical analogues for identification of T-cell precursor populations.

Cell markers (modern/old)	Cell populations							References
	HSCs	ETPs	DN1	DN2	DN3	DN4	DP	
SC-1	+	+	+	+	–	–	–	(77–82)
Sca-1	+	+	+		–	–	–	(87–94)
Sca-2	+	+	+	+	–	–	–	(87, 95–99)
CD117/c-kit	+	+	+	+	+	+	–	(43, 44, 100–102)
CD34	+	+	+	+	+	+	–	(43, 101, 102)
CD25/IL-2Rα	–	–	–	+	+	–	–	(40, 41, 85, 110)
CD44		+	+	+	–	–	–	(40, 41, 43, 102)
CD4/L3T4	–	–	–	–	–	–	+	(7, 13, 37, 86)
CD8/Lyt-2	–	–	–	–	–	–	+	(7, 13, 37, 86)
CD3/Lyt-3	–	–	–	–	–	–	+	(7, 13, 37, 86)
CD5/Lyt-1	–	–	–	–	–	–	+	(37, 86, 100, 101)
CD90/Thy-1	–	+	+	+	+	+	+	(73, 80–82)
TCRαβ	–	–	–	β	β	αβ/CD3	αβ	(13, 42, 43)
TCRγδ	–	–	–	–	γδ	γδ	γδ	(13, 42, 43)
CD8α/PNA-R	–	+	+	+	+	+	+	(7, 67, 83, 84)

DN, double negative; DP, double positive; ETPs, early T-cell precursors; HSCs, hematopoietic stem cells; PNA-R, receptor for peanut agglutinin; SC-1/Sca-1, stem cell antigen-1; Sca-2, stem cell antigen-2.

Since the anti-SC-1 immune serum was used before the development of anti-SC-1 monoclonal antibodies, it is necessary to clarify the relationship between SC-1^+^ TLPs, identified with the immune antisera and Sca-1^+^ and Sca-2^+^ TLPs, which were identified later with monoclonal antibodies. Sca-1 and Sca-2, named due to their expression by mouse bone marrow stem cells, were also evaluated for expression within the thymus. Sca-1 is expressed by cells in the thymic medulla and by some subcapsular blast cells. Sca-2 expression is limited to the thymic cortex and associated with large cycling thymic blast cells. Both Sca-1 and Sca-2 are expressed on a subpopulation of CD4^-^CD8^-^TLPs (87).

Sca-1 is currently one of the most commonly used markers of normal mouse stem cells, which was reported as a cell surface marker of HSCs (88, 89) and cells with increased tumorigenic potential (90), suggesting that Sca-1 may be an important factor in the maintenance of malignant stem cells. Sca-1 is an 18-kDa surface protein coded by the *Ly6a* gene (91). Sca-1 can interact with other proteins on the cell surface to form complex signaling pathways. This protein interacts with the TGF-β receptors and ligands, which modulate the downstream signaling in multiple organs (92). In particular, TGF-β signaling regulates Sca-1 expression, tumorigenicity, and plasticity in the mammary epithelial and cancer stem cells (88). Besides HSCs and TLPs, Sca-1 is expressed on the surface of myeloid cells and peripheral B and T lymphocytes (87–89), that is predominantly CD4^+^ T helper (Th) cells (87). Sca-1^+^ cell population may also serve as progenitors for endothelial, epithelial, and mesenchymal cells (88, 93), suggesting their high heterogeneity and plasticity, as well as multipotency, at least some of them. Sca-1 is involved in the regulation of T and B cell responses and c-kit (CD117) expression, and is believed to play roles in the differentiation, proliferation, and survival of hematopoietic and progenitor stem cells, as well as maintaining their stemness (94).

Sca-2^+^ population is the earliest known intrathymic precursor. High expression of Sca-2 is found at day 14 of mouse fetal development (95). Sca-2^+^ population is characterized by expression of an intermediate level of heat-stable antigen, a very low level of Thy-1, and a high level of CD44 antigens. It is negative for B-cell, granulocyte, macrophage, and erythrocyte markers (B220, Gr-1, Mac-1, and TER-119, respectively) (96). Within the T cell lineage, upregulation of Sca-2 expression coincides with the transition from a multipotential bone marrow stem cell to an intrathymic TLPs, suggesting an important function for Sca-2 in early thymopoiesis (97). The thymic Sca-2^+^ population is similar to bone marrow HSCs in surface antigenic phenotype and is preferentially in an activated state rather than a quiescent cell state (98). Sequence analysis of the Sca-2 protein showed that Sca-2, as well as Sca-1, is a glycosylphosphatidylinositol-anchored molecule that shares some characteristics with the members of the *Ly-6* multigene family. Sca-2 is likely identical to the mouse thymocyte shared antigen-1 (TSA-1), as the protein sequence of TSA-1 is the same as that of Sca-2 (97, 99).

Remarkably, the SC-1^+^ TLPs share properties with the Sca-1^+^ and Sca-2^+^ cell populations. Therefore, SC-1^+^ TLPs likely include both Sca-1^+^ and Sca-2^+^ TLP populations and may be presented as Sca1^+^/Sca2^+^ TLPs, which may be analogous also to DN1 ETPs or CD117^-/lo^ DN1 subtypes (100). In addition, the SC-1^+^ population includes the thymus-committed HSCs, described as the SC-1^+^Thy-1^+^ TLP2 subtype, which may overlap with early bone marrow HSCs, described as the SC-1^+^Thy-1^-^ TLP1 subtype, but yet thymus-uncommitted (73, 81, 82). CD117 receptor is the ligand for SCF, identifying HSCs, as well as the early intrathymic TLPs/ETPs (100). The thymus-committed CD117^lo^ HSC subset (likely the SC-1^+^Thy-1^+^ TLP2) is enriched with multipotent precursors and crucial for thymic regeneration and thus, also can be included in DN1 ETPs. Therefore, the SC-1^+^Thy-1^+/-^ TLP subtype is likely analogous to the combined Sca1^+^/Sca-2^+^ subtype, composing the DN1 ETP population including CD117^-/lo^ DN1 subtypes and in combination may be submitted as SC-1^+^(Sca-1^+^/Sca-2^+^)Thy-1^+/-^ CD117^+/-^ DN1 TLPs/ETPs (**Table 1**).

## 3 Proposed hypothetical mechanism of intrathymic TLPs malignant transformation

In the modern view, the DN ETPs of leukemic cells express at least one stem cell marker (CD34, CD117), and/or myeloid marker (CD11b, CD13, CD33, HLA-DR, CD65). This population consists of at least 25% leukemic blast cells and is characterized by the absence or dim expression of CD5. Additionally, ETPs lack the T-cell maturation markers CD1a and cytoplasmic CD3 (cCD3), while expressing CD7, which is one of the earliest antigens appearing on T-lymphocytes and the clinical marker of acute T-lymphocyte leukemia (101, 102).

The TC.SC-1/2.0 transformed thymic cell line was generated by multiple injections of human IL-2 into BALB/c mice. This treatment led to an increase in SC-1^+^ blast cells in the thymus, which subsequently underwent malignant transformation, possibly facilitated by a naturally occurring C-group lymphotropic retrovirus (74, 103). IL-2 administration increased the SC-1^+^ fraction up to ~10%, correlating with the emergence of cells expressing tumor-associated antigens (TAA) and clonogenic potential, absent in untreated mice (104).

**Figure 1:**
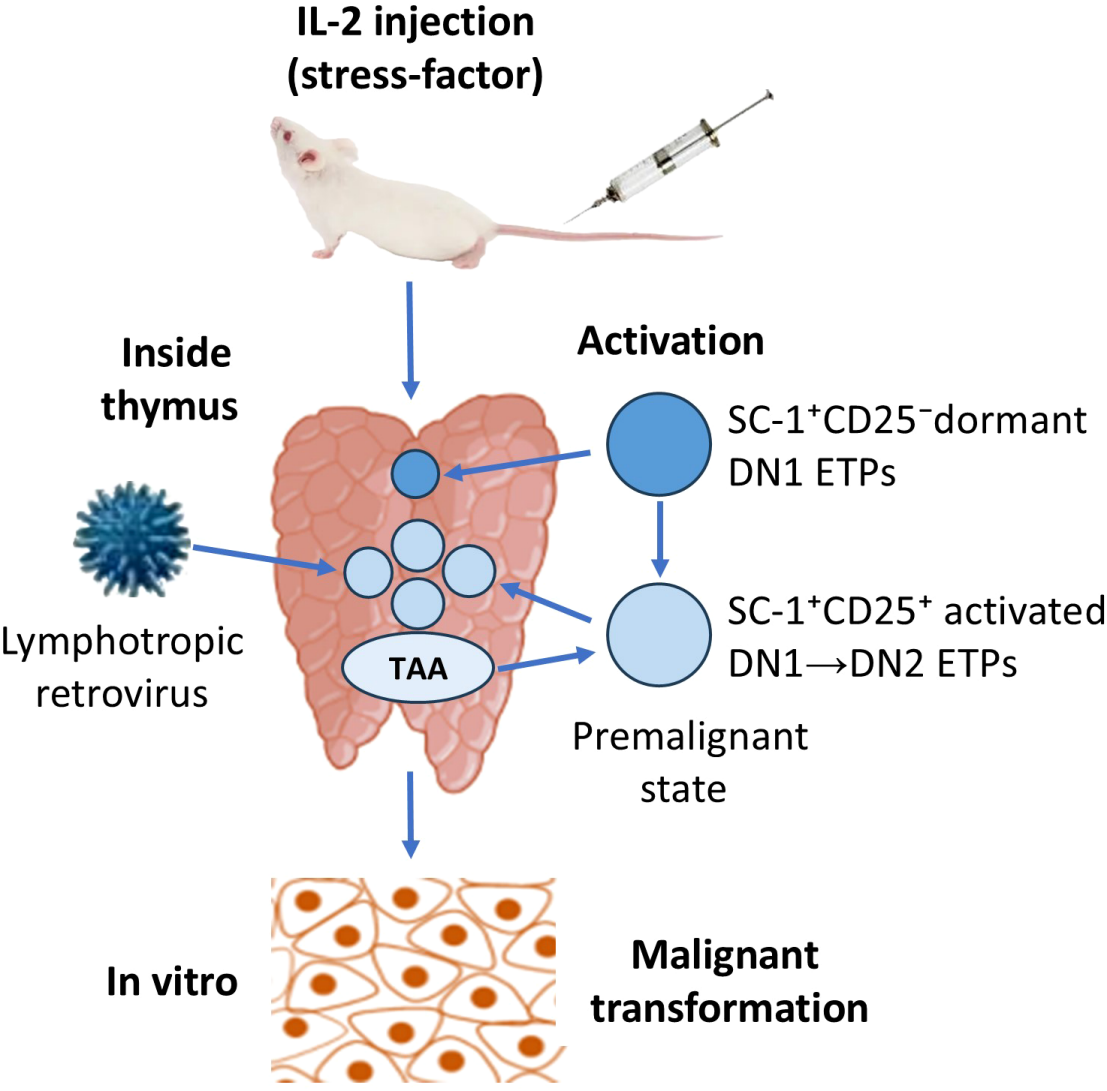
Assumed mechanism of malignant transformation of early T-cell precursors (ETPs) in BALB/c mice. Injection of human interleukin-2 (IL-2) acts as a false-activating antigenic stress-signal on dormant double-negative T-lymphocyte precursors (TLPs) stage 1 (DN1 ETPs), which are negative for IL-2 receptor (CD25). This activation induces the expression of high-affinity IL-2 receptors and abnormal accumulation of activated SC-1^+^CD25^+^ TLPs in the thymus on the transition stage from DN1 to DN2 TLPs (DN1→DN2 ETPs). The local presence of lymphotropic retroviruses may promote the infection of these cells and the emergence of tumor-associated antigens (TAA), indicating a premalignant state, which can lead to malignant transformation under relevant conditions.

These observations, together with the literature data on spontaneous and chemically induced thymic tumors in AKR mice (105–108), as well as data on acute T-cell leukemia in human (101, 102) suggest that the thymic microenvironment plays a critical role in neoplastic transformation of Thy-1^+^SC-1^+^/Sca-1^+^ TLP2 (likely CD117^+/-^ CD7^+^ ETPs), targeting them in the transition state from non-activated SC-1^+^/Sca-1^+^CD44^+^CD25^-^ DN1 to SC-1^+^/Sca-1^+^CD44^+^CD25^+^ activated DN1 state, at which they possibly expressing also Sca-2, before become CD44^+^CD25^+^ DN2 subtype. Lymphotropic retroviruses may contribute to the transformation of susceptible cells at a sufficient viral load in the thymic niche, as well as an adequate quantity of these CD44^+^CD25^+^ DN1 cells (**Figure 1**).

This suggestion is supported by the observation that transformed TLPs in AKR mice appeared only after thymic migration from bone marrow, with no transformation detected in marrow-resident cells (105, 106). This unique intrathymic niche effect is potentially mediated by stromal-epithelial signals, local cytokines, and cell–cell contacts. At this, the IL-2/IL-2R signaling appears also critical for the expansion of immature radioresistant triple-negative (CD3^-^CD4^-^CD8^-^) intrathymic SC-1^+^ TLPs for post-irradiation regeneration, likely via receptor upregulation, which activates downstream STAT5, PI3K/AKT, and MAPK pathways that promote TLP survival and proliferation (52).

From a mechanistic perspective, SC-1^+^ (Sca-1^+^/Sca-2^+^) DN1-DN2 TLPs may activate anti-apoptotic programs through Bcl-2 and Mcl-1, stress-response pathways including p53/ATM and ATF-4, and antioxidant defenses such as SOD and catalase, collectively supporting survival under genotoxic or oxidative stress (52, 56, 59, 60). These pathways likely confer resilience during irradiation or chronic inflammatory stress, explaining the accumulation of TLPs (likely CD34^+^/CD117^+/-^ ETPs) in the thymic oncogenesis model. Furthermore, split-dose irradiation, used in cancer therapy, can induce virus-independent malignant transformation of DN TLPs (ETPs), which may be accumulated due to radiation-induced maturation arrest at the triple-negative CD3^-^CD4^-^CD8^-^ stage (109). Constitutive secretion of IL-7 and SCF by thymic epithelial cells may further support survival and expansion, potentially facilitating radiation-induced leukemogenesis (7, 110). These observations integrate historical and modern knowledge, providing a framework for understanding thymic TLP transformation and highlighting targets for further experimental validation.

## 4 Phenotype profile and cell diversity of transformed TC.SC-1/2.0 thymic stem cell line

In our studies, the TC.SC-1/2.0 thymic cell line was characterized as L3T4^-^Lyt2^-^ (CD4^-^CD8^-^) transformed TLPs. This was supported by co-expression of the stem cell marker SC-1 and the T-cell marker Thy-1, along with TAA, in more than 95% of cells during early culture stages (days 80-630) (67). At later stages of culture stabilization, the proportion of Thy-1^+^ cells decreased to 18%, and TAA^+^ cells to 29%, whereas SC-1 expression remained stable (104). Conversely, PNA-R expression was initially detected in 49% of TC.SC-1/2.0 cells and dropped to 12% after treatment with the thymic hormone thymosin (67), confirming the immature and heterogeneous nature of the cell population. In the stabilized culture, PNA-R expression increased to 98%, and SC-1 remained at 94% (104).

Importantly, TC.SC-1/2.0 cells expressed IL-2R and responded to IL-2 stimulation *in vitro*, features characteristic of activated triple-negative (CD3^-^CD4^-^CD8^-^) TLPs (52) and mature CD4^+^ or CD8^+^ thymocytes after activation (111, 112). However, IL-2R expression and proliferative responses varied with the culture stage after reseeding, correlating with the level of spontaneous proliferation. These oscillations reflect cyclical processes of cell maturation, differentiation, and activation that occur within the TC.SC-1/2.0 population between days 1–4 cultures after reseeding (104).

The single-cell clonal analysis revealed that the major pool of SC-1^+^PNA^+^Thy-1^-^CD4^-^CD8^-^ TLPs, corresponding presumably to the activated CD117^+/-^ Sca-1^+^ DN1 ETPs, remained predominant (about 75-80%) and stable in the composition of the TC.SC-1/2.0 cell line. The remaining cells (20-25%) composed the subpopulations of thymocytes at different stages of maturation, preferably, the CD4^+^ T-cell lineage of development (67, 104). This heterogeneity demonstrates the self-renewing potential of TC.SC-1/2.0 cell line and its ability to undergo cycling differentiation preferably along the CD4^+^ T-helper pathway.

The expression of immunoglobulin receptors characteristic of B cells or Fc receptors (FcR) typical for macrophages, B cells, dendritic cells (DCs), granulocytes, innate lymphoid cells type 3 (ILC3), natural killer T (NKT) cells, or CD8^+^ T cells (113, 114) was not detected in TC.SC-1/2.0 cultures. Moreover, the cells lacked cytolytic activity against mouse thymocytes or ^51^Cr-labeled YAC-1 lymphoma targets (67). These data confirm the immature T-lineage identity of the TC.SC-1/2.0 cell line (**Table 2**).

**Table 2:** Expression of surface markers by the TC.SC-1.2/0 cell line during *in vitro* culturing.

Cell marker and specificity		TC.SC-1/2.0 cell line			BALB/c thymocytes*	
Marker	Specificity	Analyses number	Positive cells number (%)	Marker expression intensity (conventional units)	Positive cells number (%)	Marker expression intensity (conventional units)
Thy-1 (CD90)	HSCs, TLPs, thymocytes, T cells, NKT, DCs	11	18 ± 0.8	59 ± 1.2	96	173
L3T4 (CD4)	Thymocytes, Th cells, Tregs, NKT	13	24 ± 0.6	42 ± 0.9	94	45
Lyt-1 (CD5)	Thymocytes, T and B cells	8	20 ± 1.0	36 ± 0.8	82	40
Lyt-2 (CD8)	Thymocytes, CTL, NKT, DCs	9	5 ± 0.3	42 ± 0.5	83	67
Lyt-3 (CD3)	Thymocytes, CD8^+^T cells, NKT	9	2 ± 0.1	46 ± 0.7	88	76
SC-1** (Sca-1/2?)	HSCs, TLPs	8	94 ± 0.5	191 ± 3.0	0	-
PNA-R***	TLPs, CD4^+^CD8^+^ thymocytes	7	98 ± 0.5	121 ± 3.5	94	123
TAA****	N/A	4	29 ± 3.3	153 ± 1.1	0	-
IL-2R^#^ (CD25)	TLPs, thymocytes, T cells	-	+	Absorption, proliferation	+	Proliferation
FcR	B cells, macrophages, DCs, granulocytes, CD8^+^ T cells, ILC3, NKT	4	0	-	-	-
IgR	B cells	4	0	-	-	-

Established phenotype of TC.SC-1/2.0 cell line: SC-1^+^Thy-1^+/-^PNA^+^CD25^+^CD4^-^CD8^-^ Presumably DN1→DN2 TLPs

*Data from one analysis. **Stem cell antigen 1 – identified by antiserum obtained by immunization of rabbits with mouse brain and exhausted with mouse thymocytes (78, 79). ***Receptor for peanut agglutinin. ****Tumor-associated antigen – identified with antiserum obtained by immunization of syngeneic BALB/c mice with the TC.SC-1/2/0 cell line. ^#^Receptor for interleukin-2 – identified by absorption with TC.SC-1/2.0 cell line of IL-2-containing supernatants obtained from human (Jurkat) and mouse (EL-4) cell lines, and then analyzed in the co-stimulating proliferative test with mouse thymocytes. Modified from Shichkin et al., 1988 (67) and Shichkin, 1990 (104). No permission required.

## 5 Growth specificity of TC.SC-1/2.0 cell line *in vivo*

Continued studies demonstrated that the stable *in vitro* growth of the TLP-derived TC.SC-1/2.0 cell line required the production of a previously unidentified autocrine growth factor, designated THGF (51, 53, 72, 75, 76). Remarkable, intraperitoneal injection of TC.SC-1/2.0 cells into syngeneic BALB/c mice resulted in the formation of ascitic tumors, while some cells migrated into the thymus and developed thymomas. Furthermore, cell lines established from these tumors displayed contrasting properties (**Table 3**).

**Table 3:** Opposite properties of thymus-derived and ascites-derived sublines of the TC.SC-1/2.0 cell line.

Cell line	SP (cpm)	Proliferative response (SI) to:					
		THGF	IL-2	PHA	THGF + PHA	IL-2 + PHA	THGF + IL-2 + PHA
TC.SC-1/2.0-Th*	1269±57	1.4±0.3	3.1±0.5	1.0±0.1	1.3±0.3	2.3±0.6	5.4±0.9
TC.SC-1/2.0-As**	905±30	5.4±1.0	1.8±0.4	0.9±0.1	4.1±0.7	1.1±0.1	10.5±1.5
Thymocytes	184±10	11.3±1.1	2.0±0.3	0.9±0.1	8.7±1.0	15.3±1.3	25.3±1.5
Properties/state		TC.SC-1/2.0		TC.SC-1/2.0-As**		TC.SC-1/2.0-Th*	
THGF production		Yes		No		Yes	
Spontaneous proliferation *in vitro*		High		Very low		High	
Dividing *in vitro*		Yes		No		Yes	
Inactive (dormancy)		No		Yes		No	
Proliferative response to THGF		No		Yes		No	
Proliferative response to IL-2		Yes		No		Yes	
Proliferative response to PHA		No		No		No	
Proliferative response to THGF+PHA		No		Lower than to THGF		No	
Proliferative response to IL-2+PHA		Lower than to IL-2		No		Lower than to IL-2	
Proliferative response to THGF+IL-2+PHA		Higher than to IL-2		Higher than to THGF		Higher than to IL-2	
Presumable TLP subtype		DN1→DN2 TLPs		DN1 (dormant)		DN1→DN2 (activated)	

TC.SC-1/2.0 cells were injected intraperitoneally in syngeneic BALB/c mice. *TC.SC-1/2.0-Th subline was obtained from cells that migrated into the thymus and formed a thymoma. **TC.SC-1/2.0-As subline was obtained from cells that formed an ascites tumor. SP, spontaneous proliferation; SI, stimulation index; THGF, thymocyte growth factor; PHA, phytohemagglutinin. Combined and modified from Protsak et al, 1989 (50), Shichkin et al., 1988 (67), Shichkin et al., 1988 (72), and Shichkin, 1990 (104). No permission required.

The cell line derived from a thymoma (TC.SC-1/2.0-Th) produced THGF, proliferated actively, and was unresponsive to exogenous THGF but responded to IL-2. These characteristics match those of activated TLPs (DN1→DN2 stage) and the original TC.SC-1/2.0 cell line. In contrast, the cell line established from an ascitic tumor (TC.SC-1/2.0-As) neither produced THGF nor responded to IL-2, exhibited slow spontaneous proliferation, but responded strongly to exogenous THGF, especially in combination with IL-2 (104), likely presenting dormant TLPs similar to those which are target cells for THGF within freshly isolated thymocyte populations.

Since the thymus allows entry only to early intrathymic TLPs and committed SC-1^+^Thy-1^+^CD4^-^CD8^-^ bone marrow HSCs (similar to CD117^+/-^ DN1 ETPs), stimulated by thymic hormones such as thymosin, but restricts the entry of mature T cells (13, 49, 81), these findings support the primary stem-cell nature of the THGF-producing TC.SC-1/2.0 cell line. Its base phenotype likely corresponds to activated SC-1^+^Thy-1^+/-^PNA^+^CD25^+^CD4^-^CD8^-^ DN1→DN2 TLPs. Moreover, these findings indicate both autocrine and paracrine functions of THGF, as well as the symbiotic coexistence of at least two interacting cell populations within the TC.SC-1/2.0 cell line composition.

Interestingly, spontaneous and induced thymic tumors in AKR mice also contain cells with varying degrees of differentiation and thymic tropism (105, 108), similar to the TC.SC-1/2.0 TLP line, suggesting common targets and transformation mechanisms underlying lymphoid-type thymic tumors across different mouse strains.

In contrast to intraperitoneal injection, subcutaneous injection of the TC.SC-1/2.0 cell line in syngeneic mice produced local transplantable solid tumors without migration into thymus, when they were returned to culture *in vitro* (104), confirming their transformed nature.

Notably, the phenotypic heterogeneity of TC.SC-1/2.0 cell line correlated with karyotypic heterogeneity. Particularly, the major cell population remained stable with a modal class of 48 chromosomes, while minor populations demonstrated a chromosomal range from 42 to 52 (the normal mouse karyotype is 40 chromosomes) (103, 104).

This phenotypic and karyotypic heterogeneity suggests creating an *in vitro* microenvironment that mimics intrathymic conditions and supports complementary autocrine and paracrine interactions within the TLP sublines. Considering the presence of two cell populations with opposing THGF secretion and responsiveness, it is plausible that during TC.SC-1/2.0 cell line evolution a dynamic cooperation emerged between activated THGF-producing DN1-DN2 SC-1^+^CD25^+^ TLPs with high proliferative potential and autocrine THGF utilization, and DN1 SC-1^+^ CD25^-^ dormant TLPs, activated by THGF in a paracrine manner and maintained as multipotent stem cells. These DN1 cells may subsequently give rise to other subpopulations, progressing through DN2, DN3, and DN4 TLP stages up to DP CD4^+^CD8^+^, and to limited SP thymocytes, which may reflect the abnormal differentiation patterns of transformed TLPs or the absence of sufficient *in vitro* microenvironmental signals required for conventional cell maturation and differentiation.

## 6 Peculiarities of THGF production by TC.SC-1/2.0 cell line

Remarkably, the cells of TC.SC-1/2.0 line were capable of producing THGF spontaneously, and its production was markedly enhanced by γ-irradiation, but not by stimulation with mitogens. Among the tested irradiation doses (3–24 Gy), only doses in the range of 10–15 Gy (optimally 12 Gy) strongly activated THGF production (50, 53, 72, 104) (**Table 4**, Exp. 1, 2). At this, both spontaneous and irradiation-induced THGF secretion by TC.SC-1/2.0 cell line depended on the culture age and cell cycle phase after passage and transfer from serum-containing to serum-free medium. The spontaneous THGF production was minimal in young actively proliferating 1-day cultures. Maximum THGF secretion occurred in 2-day cultures, corresponding to the mature growth phase at the peak cell density. THGF levels declined sharply in 3-day (aging) cultures, and THGF production was virtually absent in 4-day degenerating cultures (53, 72, 104) (**Table 4**, Exp. 4).

**Table 4:** Spontaneous and induced production of THGF by cell line TC.SC-1/2.0.

Inducing factor and conditions	Proliferative response of CBA mouse thymocytes in 5-day test cultures in CCM (Stimulation index, SI)	
Experiment 1. Spontaneous and induced production of THGF for 28 hours*		
Proliferative response	Without PHA (SI)	With PHA (SI)
Spontaneous production	3.4 ± 0.2	3.8 ± 0.3
PHA (1%)	3.8 ± 0.5	3.7 ± 0.4
Con A (2.5 μg/ml)	2.2 ± 0.3	2.1 ± 0.2
PMA (10 ng/ml)	2.0 ± 0.1	1.8 ± 0.1
γ-irradiation (12 Gy)	45.0 ± 1.6	47.0 ± 2.0
γ-irradiation (12 Gy) + PHA (1%)	38.2 ± 1.4	37.5 ± 1.8
Experiment 2. Dose-dependent effect of γ-irradiation on THGF production for 28 hours*		
γ-irradiation doses (Gy)	Without PHA (SI)	TC.SC-1/2.0 cell viability via 28 hours (%)**
0	0.8 ± 0.2	8.3 ± 2.5
3	5.8 ± 0.9	7.0 ± 2.0
6	6.4 ± 1.1	5.1 ± 1.5
9	15.2 ± 2.2	7.2 ± 1.8
12	21.9 ± 3.7	12.6 ± 3.3
18	8.1 ± 2.1	3.0 ± 0.4
24	5.8 ± 1.2	0.5 ± 0.1
Experiment 3. Dynamics of THGF production during 48 hours*		
Time (hours) after γ-irradiation (12 Gy)	Without PHA (SI)	TC.SC-1/2.0 cell viability during 48 hours (%)**
0	0	47.7 ± 4.8
4	2.0 ± 0.3	27.5 ± 4.3
20	42.3 ± 6.0	25.3 ± 3.3
24	45.2 ± 5.5	24.4 ± 3.2
28	47.4 ± 5.0	20.2 ± 2.7
48	42.5 ± 4.3	10.0 ± 1.5
Experiment 4. Spontaneous and irradiation-induced THGF production for 28 hours in dependence on culture age after cell reseeding*		
Time after cell culture reseeding (days)	Spontaneous production (SI)	Irradiation induced production, 12 Gy (SI)
0	0	0
1	1.2 ± 0.1	17.5 ± 1.8
2	10.4 ± 0.4	35.7 ± 2.5
3	4.5 ± 0.2	27.4 ± 2.0
4	1.3 ± 0.1	2.5 ± 0.2

*Serum-free culture medium was used for the production of THGF. **Initial cell viability consisted 80-85%. CCM, complete culture medium; Con A, concanavalin A; PHA, phytohemagglutinin; PMA, phorbol myristate acetate. Adopted and combined from Protsak et al., 1989 (50), Shichkin, 1992 (53), Shichkin et al., 1988 (72), Shichkin, 1990 (104). No permission required.

In all culture stages, the level of irradiation-induced THGF secretion substantially exceeded that of spontaneous production. This enhancement reflected the activating effect of the specific 12 Gy dose on *de novo* THGF synthesis, rather than the release of pre-formed factor from damaged or dying cells. Several observations support this conclusion: a) irradiation doses below or above 12 Gy were far less effective in inducing THGF despite comparable rates of cell death; b) the death rate of irradiated cells only slightly exceeded that of non-irradiated controls in serum-free conditions (53, 72, 104) (**Table 4**, Exp. 2).

The 48-hour dynamics of THGF secretion after irradiation also substantiated this suggestion (72, 104). Immediately after exposure, cell viability decreased dramatically, while no THGF activity was detected in the supernatant. Minimal THGF activity appeared 4 hours post-irradiation, coinciding with a further drop in cell viability. Over the next 20 hours, THGF activity in the culture medium increased sharply, reaching a maximum at 28 hours, while cell viability remained relatively constant at approximately 27%. Finally, cell viability declined by 48 hours from 80% to 10%. However, no further growth of THGF activity was observed after 28 hours of culturing (**Table 4**, Exp. 3).

Remarkably, when these irradiated cells were reseeded again into fresh serum-free medium after 24 hours of culturing, no THGF activity was detected in this new supernatant, confirming that THGF is not stored intracellularly but synthesized and secreted actively in response to irradiation (72). These data also indicate a 4-hour latent phase followed by 16 hours of active THGF synthesis and secretion, with minor continued accumulation up to 28 hours. The residual increase likely reflects incomplete synchronization of the irradiated culture before seeding. The data also suggest that approximately 30% of the TC.SC-1/2.0 cell population is active radioresistant THGF producers. Remarkably, under comparable conditions, γ-irradiation did not stimulate IL-2 secretion by the EL-4 cell line (53, 72, 104), emphasizing fundamental differences between THGF- and IL-2-producing cells, as well as between these cytokines themselves.

During the formation of the TC.SC-1/2.0 cell line, a distinct pattern emerged in the ratio between spontaneous and irradiation-induced (12 Gy) THGF secretion. THGF activity was first detected in conditioned medium on day 77 of the cell line formation. Up to approximately 550 days, spontaneous THGF production remained low but increased markedly following irradiation. With further stabilization, spontaneous THGF secretion rose progressively and eventually equaled the level of irradiation-induced production (50, 53, 72, 104). These irreversible changes reflected evolutionary stabilization and cellular diversification within the TC.SC-1/2.0 cell line, during which continuous THGF production functioned likely as a key autocrine-paracrine factor in selective cell survival and expansion.

Collectively, these findings suggest that THGF is not stored in intracellular depots but secreted constitutively during synthesis, similar to most thymic cytokines, and utilized quickly by proliferating cells, likely in autocrine - paracrine manner.

## 7 Properties of THGF

### 7.1 Biochemical characteristics and THGF effects *in vitro*

THGF activity, which is contained in the serum-free conditioned supernatant of TC.SC-1/2.0 cell line is a charge-heterogeneous acidic glycoprotein stable to heating at 56 °C and resistant to both acidic (pH 2.0) and alkaline (pH 10.0) conditions. It is precipitated by ammonium sulfate at 40–85% saturation, binds irreversibly to Con A–Sepharose, and is inactivated by trypsin (72, 75, 104).

THGF was isolated and purified from serum-free supernatants of TC.SC-1/2.0 cell cultures using ammonium sulfate precipitation, gel filtration chromatography, and HPLC. The final product, purified to 99%, was submitted by glycoprotein components with molecular weights of 22 kDa (75). This glycoprotein exhibited specific THGF activity in standard mouse thymocyte proliferation assays, with maximal responses observed on day 5-7 and did not enhance proliferation of thymocytes in the presence of mitogens similar to the crude THGF-containing supernatant (76) (**Table 5**).

**Table 5:** Biochemical characteristics and properties of THGF.

Biochemical characteristics and functional activity	Reference
Biochemical nature: 22 kDa charge-heterogeneous acidic glycoprotein	(72, 75, 104)
Stabile at: heating at 56°C, acidic (pH 2.0) and alkaline (pH 10.0) conditions	
Ammonium sulfate precipitation: at saturation of 40–85% concentration	
Con A-Sepharose binding: irreversibly	
Trypsin effect: inactivates	
Optimal and minimal activating concentration for thymocytes: 12.5-25% or 8-16 pg/ml (optimal) and 1.25-3.0% or 1-2 pg/ml (minimal), of the TC.SC-1/2.0 cell line crude supernatant and purified THGF, correspondingly	(72, 76, 104)
Specific thymocyte-stimulating activity: 17.9 units/mg and 2.85 x 10^8^ units/mg of protein in the TC.SC-1/2.0 cell line crude supernatant and purified THGF, correspondingly	(75)
Minimal and optimal preincubation time with THGF for thymocyte activation: 1 hour – minimal; 4 hours – 50% activation; 24 hours – maximal activation	(76)
THGF effects *in vitro*	Reference
Direct activating and proliferative effects on mouse thymocytes: untreated, cortisone-resistant, radioresistant, long-lived, PNA+ fraction (presumably DN1-DN3), SC-1+ fraction (presumably DN1), L3T4-Lyt-2- fraction (presumably DN1-DN3), and abolition of intact thymocytes proliferation at treatment with: anti-SC-1 antisera + complement, anti-Thy-1.2 antibodies + complement, and thymotropin	(68, 72, 73, 76, 104)
The increase of proliferative effects on mouse thymocytes in the presence of IL-1, IL-2, IL-3: unfractionated, long-lived, radioresistant (only with IL-2)	(51, 53, 104)
The absence of proliferative effect increases on mouse thymocytes in the presence of PHA, ConA, PMA: unfractionated, radioresistant, long-lived	(68, 72, 73, 76, 104)
Species-specificity of mouse thymocytes' proliferative response: mouse – respond, rat – nonresponse, human - nonresponse	(72, 104)
Seasonal fluctuations of intact thymocyte proliferative response: autumn↑, winter↑, spring↓, summer↓	(104)
Differentiation effect on thymocytes: No conversion of PNA^+^ to PNA^-^ stage	(104)
Induction of apoptosis in THGF-responding thymocytes: no	(104)
Direct activating and proliferative effects on mouse splenocytes: unfractionated, non-adhering, Ig^-^ fraction, Ig^+^ fraction (presumably IgE^+^ B cells), α_1_-thymosin-treated (no or decrease)	(76)
Direct activating and proliferative effects on mouse lymph node cells: unfractionated, α_1_-thymosin-treated (no or decrease)	(76)
Direct activating and proliferative effects on mouse bone marrow cells: unfractionated (no effect), SC-1^-^ fraction (no effect), anti-SC-1^+^ antisera + complement treated (no effect), thymotropin-treated (activation of proliferation)	(68)
THGF effects *in vivo*	Reference
Colony-stimulating activity: induction of colony-formation in spleen by administration of bone marrow SC-1^-^ Th-1^-^ cells treated *in vitro* with THGF (exo-test) and by THGF administration *in vivo* (endo-test) to lethally and sublethally irradiated CBA mice, correspondingly	(73, 76, 104)
THGF administration: stimulates the accumulation of mature PNA^-^ T cells in the thymus and their further migration to the periphery; enhances the ability of lymph node lymphocytes to mediate graft rejection; increases the serum IgE level but does not impact IgG level upon immunization with bovine serum albumin and IgM^+^ and IgG^+^ cell accumulation in the spleen upon immunization with sheep erythrocytes	(72, 104)

The optimal stimulatory concentration of purified THGF was 8–16 pg/ml, corresponding to 12.5-25% concentration of the TC.SC-1/2.0 crude supernatant, whereas doses as low as 1–2 pg/ml were sufficient to induce measurable thymocyte proliferation (76). In contrast, optimal doses for most other thymus-associated cytokines under comparable conditions ranged between 50–100 ng/ml (110, 115).

Preincubation of thymocytes with THGF for 60 minutes was sufficient to initiate proliferative activity. After 4 hours preincubation, the response reached ~50% of maximum, and full activation was developed after 24 hours of preincubation. Prolonged exposure did not enhance proliferation further (72, 76, 104).

These findings support the suggestion that THGF exhibits both mitogenic and autocrine growth-supporting properties. It means that exogenous THGF, presumably, may stimulate the synthesis of endogenous THGF, thereby sustaining the proliferation of responsive thymic cells.

THGF activity was species-specific, similar to that of most murine cytokines. In particular, THGF did not stimulate the proliferation of rat and human thymocytes. However, it was equally active toward allogeneic mouse thymocytes (72, 104).

Interestingly, the proliferative response of thymocytes to THGF showed seasonal variability. In spring and summer, proliferation developed more slowly and reached a lower intensity compared to autumn and winter (104). This effect likely reflects seasonal fluctuations in the number or activity of THGF-responsive thymic cells. While circadian rhythms are known to have an impact on the immune system and cytokine production in physiological conditions, similar seasonal dependency has not been reported for other cytokines (116–118).

### 7.2 THGF effects *in vivo*

Intraperitoneal administration of THGF-containing supernatants to mice stimulated the accumulation of mature PNA^-^ T cells in the thymus and their subsequent migration. This effect likely reflects stimulation of TLP maturation through interactions with other components of the intrathymic microenvironment, since *in vitro* THGF did not induce PNA^+^ to PNA^-^ conversion or thymocyte death. Such *in vivo* activity is consistent with the suggested physiological role of THGF in promoting and maintaining thymic regeneration following injury, particularly after irradiation (50, 72, 104).

Beyond its thymic effects, THGF enhanced the ability of lymphocytes in regional lymph nodes to mediate graft rejection and substituted for TLP2 in supporting bone marrow HSCs. This was evidenced by an increase in the colony-forming activity of the SC-1^-^ bone marrow HSC fraction and elevated endogenous spleen colony formation in sublethally irradiated mice (72, 104).

THGF administration did not influence the accumulation of IgM- or IgG-producing cells in the spleen of mice immunized with sheep erythrocytes, nor did it enhance serum IgG levels upon immunization with bovine serum albumin. However, THGF increased serum IgE levels by 1.5-2-fold (72, 104), assuming a potential contribution of THGF to the development of immediate-type hypersensitivity reactions (**Table 5**).

### 7.3 THGF effects on peripheral lymphoid cells

In additional to thymocytes, splenocytes and lymph node cells also proliferated in response to THGF. When splenocytes were separated into Ig^+^ (B cells) and Ig^-^ (T cells, macrophages, dendritic cells, neutrophils, and eosinophils) fractions, both populations were responding to THGF, though the Ig^-^ fraction exhibited a stronger proliferative response comparable to that of unfractionated cells. Removal of adherent splenocytes did not alter THGF-induced proliferation (**Tables 5**, **6**) (76), suggesting that THGF targets likely are not stromal or epithelial cells.

**Table 6:** Proliferative response of thymocytes, bone marrow cells, splenocytes and lymph node cells of CBA mice to THGF-serum free supernatants in 5-day test-cultures without co-mitogens, and colony-stimulating activity of THGF.

Experiment number and cell sources*	Cell fractions and treatments*			Stimulation index*
1. Thymus	Unfractionated			20.7 ± 2.7
	Cortisone-resistant (PNA^+^ TLPs)			22.6 ± 4.2
	PNA^+^ fraction			23.3 ± 0.3
	PNA^-^ fraction			8.1 ± 0.9
2. Thymus	Unfractionated			5.3 ± 0.4
	SC-1^-^ (DN4) fraction			1.4 ± 0.2
	L3T4^-^Lyt-2^-^ (DN1-DN3) fraction			5.0 ± 0.4
	Thymotropin-treated (SC-1^-^ DN4)			1.2 ± 0.1
3. Bone marrow	Unfractionated			0.5 ± 0.1
	SC-1^-^ fraction			0.6 ± 0.1
	Thymotropin-treated (SC-1^+^ DN1-DN2)			12.1 ± 1.2
4. Spleen	Unfractionated			7.9 ± 0.7
	IgG^-^ fraction			7.4 ± 0.5
	IgG^+^ fraction			4.3 ± 0.3
5. Spleen	Unfractionated			5.8 ± 0.6
	α_1_-thymosin-treated			4.6 ± 0.3
6. Lymph nodes	Unfractionated			2.9 ± 0.2
	α_1_-thymosin-treated			2.0 ± 0.2
7. Spleen**	Unfractionated			4.8 ± 0.4
	IgG^-^ fraction			2.9 ± 0.2
	IgG^+^ fraction			4.0 ± 0.3
Colony-stimulating activity of THGF in lethally (9,5 Gy, exotest) and sublethally (8.5 Gy, endotest) irradiated CBA mice				
Cell sources of tested supernatants	Number of colonies in spleen induced by administration of bone marrow SC-1-Thy-1- cells treated *in vitro* by the supernatant (exotest)		Number of colonies in spleen induced by the supernatant administration *in vivo* (endotest)	
	Not treated	Treated	Not treated	Treated
Bone marrow SC-1^+^Thy-1^+^ cells	0	6.5 ± 1.9	0	6.3 ± 2.9
Radioresistant thymocytes	1.2 ± 0.3	7.5 ± 0.2	ND	ND
Cortisone-resistant thymocytes	0.8 ± 0.4	5.5 ± 0.5	1.2 ±0.7	4.0 ± 0.3
L3T4^-^Lyt-2^-^(CD4^-^CD8^-^) thymocytes	0	19.2 ± 5.5	ND	ND
TC.SC-1/2.0 cell line	0	6.7 ± 3.6	0.6 ± 0.5	2.5 ± 0.5
Pure THGF	1.0 ± 0.1	5.7 ± 1.3	0	12.4 ± 4.7
WEHI-3 cell line	0	6.2 ± 1.5	0.6 ± 0.5	3.0 ± 0.6
Jurkat cell line	0.4 ± 0.1	0.5 ± 0.3	1.8 ± 1.0	1.6 ± 0.8

*Proliferative activity in response to THGF. **Purified THGF was used in the experiment. ND, not detected. Combined and adopted from Protsak et al., 1989 (68), Yarilin at al., 1990 (73), Talaev et al., 1991 (76), Shichkin, 1990 (104). No permission required.

Thus, activation of THGF-producing cells may presumably contribute to the development of stress-induced, IgE-mediated allergic responses through the B-cell–activating properties of THGF.

### 7.4 Colony-stimulating activity of THGF

Most HSCs in the bone marrow do not express the Thy-1 antigen, or its expression is minimal. However, a subset of SC-1^-^ colony-forming cells can display high-density surface Thy-1 expression (78, 80, 119). When SC-1^+^Thy-1^-^ bone marrow precursors, similar to the intrathymic TLP1, are exposed to thymic hormones, they begin to express Thy-1 without losing SC-1, thus forming a Thy-1^+^SC-1^+^ population similar to intrathymic TLP2. These TLP2 cells exhibit enhanced helper activity in hematopoietic colony formation compared with TLP1 (79, 82). The colony-stimulating activity associated with this TLP2 population has been shown to result from the production of a specific colony-stimulating factor (CSF) by Thy-1^+^SC-1^+^ bone marrow TLP2 cells (71, 73).

Evidence supporting the CSF-producing capacity of Thy-1^+^SC-1^+^ TLP2 thymocytes was obtained in our experiments. CBA mice were treated either with hydrocortisone (250 μg per gram body weight) or total-body γ-irradiation (4 Gy). Thymic L3T4^-^Lyt-2^-^ cells, isolated by selective cytolysis using anti-L3T4 and anti-Lyt-2 monoclonal antibodies with rabbit complement, produced high levels of CSF activity. Elimination of SC-1^+^ cells with anti–SC-1 serum and complement abolished CSF production. All functional properties of this CSF activity were confirmed in spleen colony formation assays in irradiated mouse recipients (73) (**Tables 5**, **6**).

Remarkably, the supernatant of the TC.SC-1/2.0 cell line, as well as purified THGF, stimulated colony formation by SC-1^-^Thy-1^-^ bone marrow cells, like supernatants derived from Thy-1^+^SC-1^+^ bone marrow TLP2 cells (73, 76). A comparable effect was observed with supernatants from the IL-3-producing WEHI-3 cell line, but not with recombinant human IL-2 or supernatants from IL-2-producing human Jurkat cell line, which contain a mixture of cytokines (**Table 6**).

Conversely, supernatants from normal or transformed Thy-1^+^SC-1^+^ TLPs of either bone marrow or thymic origin, including TC.SC-1/2.0 cells did not support the growth of IL-3- or IL-2-/IL-4-dependent cell lines (73). Thus, although Thy-1^+^SC-1^+^ TLP-derived colony-stimulating activity and IL-3 show functional similarities, they are not identical. Furthermore, THGF is likely similar to the CSF, described by Yarilin’s group, which is produced by Thy-1^+^SC-1^+^ bone marrow TLP2 cell population and TC.SC-1/2.0 TLP cell line (71, 73).

In summary, beyond its proliferative effects on thymic TLPs, splenocytes, and lymph node cells, THGF also stimulates hematopoietic colony formation in the spleens of irradiated mice. These data suggest that bone marrow and early intrathymic TLPs (presumably activated DN1→DN2) generate THGF-like activity that functions as a CSF for HSCs. Consequently, bone marrow TLPs and intrathymic THGF-responsive TLPs probably represent interdependent stem/progenitor cell populations that operate in coordination during thymic regeneration under extreme physiological stress.

### 7.5 Identification of irradiation-induced THGF-like activity produced by thymocytes

It was previously shown that radioresistant SC-1^+^ TLPs are accumulated in the mouse thymus on days 2–5 after sublethal total-body irradiation, corresponding to the early phase of thymic recovery (49, 79). The established TC.SC-1/2.0 cell line exhibited a phenotype similar to intrathymic SC-1^+^ TLPs, and its THGF-producing activity was associated with γ-irradiation. Therefore, we examined supernatants of thymic cell cultures from irradiated mice for the presence of THGF-like activity.

As expected, thymocytes isolated on days 2–12 after 4 Gy sublethal γ-irradiation of CBA mice demonstrated the presence of THGF-like activity in the supernatants of 28-hour serum-free cultures. This activity was yet more essential upon re-irradiation of these thymocytes *in vitro* at 12 Gy, 2 days after *in vivo* irradiation. Moreover, THGF-like activity was also detected in the supernatants of thymocytes irradiated only *in vitro* with a dose of 12 Gy. However, at a markedly lower level than that observed in pre-irradiated mice (53, 104) (**Table 7**).

**Table 7:** Identification of THGF-like activity in serum-free supernatants obtained at 28-hour culturing of thymocytes isolated from CBA mice (Experiment 1) and BALB/c mice (Experiment 2) irradiated with a dose of 4 Gy and 3.5 Gy, respectively, and comparison of THGF activity with other relevant cytokines.

Terms after irradiation of mice	Number of mice in experiments and thymocytes after irradiation (median)		Proliferative response of intact CBA mouse thymocytes in 5-day test cultures to supernatants obtained as a result:			
			Spontaneous production (direct test)		Thymocytes irradiated *in vitro* (12 Gy) (direct test)	
Experiment 1	10 mice		cpm	SI	cpm	SI
2 days	1 x 10^6^		741	5.2 ± 0.5	2049	14.4 ± 1.7
5 days	0.2 x 10^6^		923	6.5 ± 0.7	414	2.9 ± 0.1
12 days	35 x 10^6^		611	4.3 ± 0.4	598	4.2 ± 0.3
No irradiation	70 x 10^6^		184	1.3 ± 0.1	383	2.7 ± 0.3
Experiment 2	5 mice		Without PHA (SI)	With PHA (1%) (SI)	Without PHA (SI)	With PHA (1%) (SI)
2 days	7.7 x 10^6^		3.8	1.8	0.9	4.1
5 days	3.7 x 10^6^		2.9	2.4	1.1	8.2
12 days	27 x 10^6^		3.3	2.8	1.4	7.0
22 days	48 x 10^6^		3.0	2.0	1.4	7.5
No irradiation	180 x 10^6^		1.0	1.0	0.8	0.7
Comparison of THGF activity with relevant cytokines						
Cytokine-contained medium		Thymocytes		CTLL-2 (IL-2/IL-4-dependent)	32D.c1-23 (IL-3-dependent)	STh-870 (THGF-dependent)
		Without PHA (SI)	With PHA (SI)			
THGF-CCM		21.2 ± 1.8	37.2 ± 2.5	5.9 ± 0.7	2.5 ± 0.4	11.8 ± 1.3
THGF-ICM		11.3 ± 1.1	8.7 ± 1.0	0.9 ± 0.1	1.0 ± 0.1	5.7 ± 0.5
IL-1 (J-774-CCM)		0.8 ± 0.2	12.7 ± 1.1	2.7 ± 0.3	1.1 ± 0.2	1.3 ± 0.1
IL-2 (EL-4-ICM)		2.0 ± 0.3	15.3 ± 1.3	140.7 ± 5.2	3.9 ± 0.4	1.3 ± 0.1
IL-3 (WEHI-3-CCM)		0.6 ± 0.1	3.0 ± 0.3	6.3 ± 0.6	45.0 ± 2.7	1.2 ± 0.1
Purified THGF and recombinant cytokines*		Thymocytes				
		Without PHA (SI)	With PHA (SI)	Anti-IL-2R mAb 7D4	Anti-IL-4 mAb 11B11	Anti-TSA serum
THGF (8 pg/ml)		3.2	2.06	3.1	2.9	3.0
IL-2 (100 ng/ml)		60.9	992	NT	NT	NT
IL-4 (50 ng/ml)		3.6	371	NT	NT	NT
IL-7 (50 ng/ml)		4.7	0.7	NT	NT	NT
IL-9 (100 ng/ml)		2.2	13.7	NT	NT	NT
SCF (25 ng/ml)		3.2	2.2	NT	NT	NT
GM-CSF (25ng/ml)		1.7	3.1	NT	NT	NT

CCM, complete culture medium; ICM, incomplete culture medium; NT, not tested; PHA, phytohemagglutinin; TSA, tumor-specific antigen; SI, stimulation index. *Showed for doses with maximal proliferative effect in the tested range 0.25-16 pg/ml of THGF and 12.5-100 ng/ml of recombinant cytokines. Combined and adopted from Shichkin, 1992 (53), Talaev et al., 1991 (75), Shichkin, 1990 (104), Shichkin and Durum, 2000 (124). No permission required.

These findings emphasize the essential role of the intrathymic microenvironment in mediating full irradiation-induced responses and assume a key function of THGF or THGF-like activity in post-irradiation thymic regeneration at the level of early DN TLPs.

### 7.6 THGF in the context of thymic cytokine biology

The biological activity of THGF was first identified by us in 1984 through its direct mitogenic effect on freshly isolated CBA mouse thymocytes, cultured for five days in the absence of mitogens or additional co-stimulatory signals. This activity was initially described as IL-2-like based on its functional proliferative effect, rather than molecular identity. Subsequent experiments demonstrated that classical polyclonal mitogens, concanavalin A (Con A), phytohemagglutinin (PHA), and phorbol myristate acetate (PMA), did not potentiate the proliferative effect of THGF (50, 53, 72, 76, 104). This observation provided early evidence that THGF is not functionally identical to IL-1, IL-2, IL-3 or IL-4, which typically act as co-mitogenic or activation-dependent cytokines. Importantly, THGF-induced proliferation occurred independently of co-mitogenic IL-2-dependent classical T-cell activation pathways, suggesting a fundamentally different mode of thymocyte regulation.

It is now well established that γ-irradiation induces the expression of a broad spectrum of cytokines and growth factors, including IL-1, IL-3, IL-4, IL-6, IL-10, IL-12, IFN-γ, stem cell factor (SCF), G-CSF, GM-CSF, TNF-α, and TGF-β, as demonstrated in multiple *in vivo* and *in vitro* models (120–123). Several of these molecules, most notably IL-7, SCF, IL-2, and IL-4, play critical roles in thymopoiesis and therefore represent logical candidates for comparison with THGF. Moreover, given the colony-stimulating activity of THGF, IL-3 and GM-CSF can also be added to this group, as well as IL-22, considering its role in thymic tissue regeneration.

Among thymopoietic cytokines, IL-4, IL-7, and SCF are constitutively produced within the thymus under physiological conditions (7, 110). However, radiation-induced upregulation has been convincingly demonstrated only for IL-4 and SCF. Following total-body irradiation (9 Gy, ^137^Cs), SCF was detected in mouse plasma on days 1 and 4 post-irradiation, whereas IL-4 appeared in plasma at approximately 6 hours post-exposure (122, 123), demonstrating the properties of systemic cytokines. In contrast, THGF displayed kinetics and bioavailability consistent with a locally acting thymic factor rather than a systemic cytokine.

Direct comparative analyses revealed that THGF possesses unique functional properties *in vitro* and *in vivo* distinct from IL-1, IL-2, IL-3, IL-4, IL-7, IL-9, SCF, and GM-CSF (50, 53, 72, 76, 104, 124). Although IL-7 and SCF exhibited the closest functional resemblance to THGF in terms of the intact thymocyte proliferation activation (125–128), the effective concentration of THGF required for maximal stimulation was dramatically lower - approximately 6,250-fold lower than IL-7 and 3,125-fold lower than SCF. For these cytokines, optimal concentrations were in the range of 25–50 ng/ml. In contrast, maximal THGF-induced proliferation was achieved at 12.5–25% of the TC.SC-1/2.0 culture supernatant (72) or 8–16 pg/ml of purified THGF (76) (**Table 7**).

Crucially, THGF-mediated thymocyte proliferation was not inhibited by monoclonal antibodies against IL-2Rα (CD25) or IL-4, providing definitive evidence that THGF signaling does not engage canonical IL-2 or IL-4 receptor pathways (7, 53, 72, 104). These findings support the suggestion that THGF operates via a distinct receptor system.

Notably, IL-7, initially described in 1988 as a pre-B-cell growth factor (129), was later shown to have properties similar to IL-2 and IL-4 in the proliferation of mouse thymocytes, primarily in combination with PHA (115). In these systems, IL-2 and IL-7 also acted as direct mitogens, whereas IL-4 required additional co-mitogenic stimulation. Importantly, the optimal concentrations of IL-2, IL-4, and IL-7 in these experiments exceeded those of THGF in our analogous experiments by more than three orders of magnitude (72, 76, 115), underscoring the exceptional potency of THGF.

In addition, THGF is functionally distinct from IL-1, IL-3, and IL-9, which in our experiments exhibited co-stimulatory activity in the presence of PHA or Con A but were not active in the absence of mitogens (IL-1 and IL-3) or demonstrated low activity (IL-9) (51, 72, 76, 104, 124) (**Table 7**).

From a modern perspective, the extraordinarily low effective dose of THGF, combined with the high sensitivity of target thymocytes, suggests a short-distance paracrine or autocrine mode of action, characteristic of niche-restricted thymic cytokines, consistent with the diversification of THGF-producing cells within a single clone (67, 104). Such signaling behavior is now recognized as a hallmark of tissue-specific thymic cytokines, such as IL-2, IL-4, IL-7, IL-15, and TGF-β, that govern cellular diversification, survival, and lineage commitment within confined microanatomical compartments (130–135). This also implies the existence of high-affinity and high-specific THGF receptors, distinct from those used by analogous cytokines.

Among cytokines described more recently, IL-22 exhibits certain functional parallels with THGF, particularly in its role as a tissue-restricted regulator, acting at epithelial–stromal interfaces in post-radiation thymic regeneration (136–139). However, IL-22 differs fundamentally from THGF with respect to cellular sources, target populations, molecular weight, and biological context (**Table 8**).

**Table 8:** THGF versus the most comparable cytokines in the mouse thymus.

Indicator/Cytokine	THGF	IL-7	SCF	GM-CSF	IL-2	IL-3	IL-4	IL-22
Mol. weight (kDa)	22	17.4/25	18.5	14-34	15.5	15-20	15	16-18/28-40
Receptor	Unknown (high-affinity)	IL7Rα / IL2Rγ	c-kit	GM-CSF-Rαβ	IL2Rαβγ	IL-3Rα/β	IL4Rα/ IL2Rγ	IL22R1 / IL10R2
Producers	Presumably DN1→DN2	TEC, MSC, DC	TEC, MSC, DC, Mac	Mac, TC, MSC, EC, NK, Fb	TC, DC, NK, NKT	TC, Mac, NK	Th2, Bas, MC	γδ T cells, Th17, ILC3
Secretion	Primarily induced by stress factors	Constitutive / Induced	Constitutive	Constitutive	Induced	Induced	Induced	Induced by stress factors
Irradiation effect on production	Activation and increase	No	Activation	No	No	No	Activation	No direct effect
Target cells in thymus	Presumably DN1 dormant→DN2 THGF-activated	DN2-DN4	DN1-DN2	DN1-DN2	DN2-DN3	DN1-DN2	DN2-DN3	TECs
Radioresistant long-lived cells (response)	Yes/High	No	Probably No/Low	Probably Yes/Low	No	No	No	Unknown
Effect on TLPs	Activation and proliferation	Survival and proliferation	Survival and proliferation	Growth support	Growth and differentiation	Growth support	Growth and differentiation	No
Direct effect on intact thymocytes	Yes Very low doses	Yes High doses	Yes High doses	No or very Low High doses	Yes High doses	No	Yes High doses	No
Co-mitogenic effect	No	No	No	Yes	Yes/High	Yes	Yes/High	No
HSC colony-formation	Yes	No	Yes	Yes	No	Yes	No	Unknown
Action mode	Autocrine / paracrine	Autocrine / paracrine	Autocrine / paracrine	Autocrine / paracrine	Autocrine / paracrine	Autocrine / paracrine	Autocrine / paracrine	Paracrine / autocrine
References	(50, 51, 53, 67, 68, 72, 73, 75, 76, 104, 124, 145)	(7, 8, 110, 121, 123, 125–127, 151)	(7, 8, 110, 121, 123, 126, 128)	(7, 8, 110, 121, 123, 126)	(52, 110, 112, 121, 126, 144)	(7, 8, 110, 121, 123, 126)	(7, 8, 12, 120–123, 126)	(7–9, 136–139)

DN, double-negative; DC, dendritic cells; EC, endothelial cells; Fb, fibroblasts; HSC, hematopoietic stem cells; ILC, innate lymphoid cells; Mac, macrophages; MC, mast cells; MSC, mesenchymal cells; NK, natural killers; NKT; natural killer T cells; SFs, stress factors; TC, T cells; TEC, thymic epithelial cells; THGF, thymocyte growth factor; TLPs, T-lymphocyte progenitors.

Thus, while THGF may represent a conceptual analogue within the broader framework of niche cytokines, such as IL-2, IL-4, IL-7, IL-15, IL-22, and colony-stimulating growth factors, such as IL-3, SCF, and GM-CSF, they themselves cannot replace the unique biological profile of THGF (**Table 8**). However, based on the properties of TC.SC-1/2/0 cell line and Sca-1 biology, we cannot exclude the possibility that THGF may be a soluble form of Sca-1, and the Sca-1/Sca-2 receptor complex may act as an autocrine/paracrine pathway for the self-regulation of THGF-producing cells.

## 8 Identification and properties of intrathymic THGF-responding cells

### 8.1 Phenotyping identification of THGF-responding thymocytes

Since the TC.SC-1/2.0 cell line expressed both SC-1 and PNA receptors and utilized THGF as an autocrine-paracrine growth factor, we hypothesized that THGF target cells in the thymus may share a similar phenotype and properties. To identify these target populations, the responsiveness to THGF was evaluated in different fractions of thymocytes, including cortisone-resistant, PNA^+^, PNA^-^, SC-1^+^, and L3T4^-^Lyt2^-^ subsets (68, 73, 76, 104) (**Table 6**).

Following hydrocortisone administration, only about 10% of thymocytes remained in the thymus, consisting predominantly of PNA^-^ (97%) and a minor fraction of PNA^+^ (3%) cells. Despite this reduction, the residual cortisone-resistant thymocytes retained their responsiveness to THGF. At this, the proliferative response of the PNA^+^ fraction was comparable to that of PNA^+^ thymocytes from intact mice and unfractionated thymocytes, suggesting that exactly PNA^+^ thymocytes are targets for THGF. The apparent THGF responsiveness of the PNA^-^ population was likely due to contamination of this by approximately 10% PNA^+^ thymocytes (68, 104) (**Table 6**). Furthermore, injection of THGF into sublethally irradiated mice stimulated the accumulation of immature SC-1^+^PNA^+^CD4^-^CD8^-^ thymocytes and enhanced their responsiveness to THGF and IL-2 *in vitro* (51, 53).

The early intrathymic PNA^+^CD25^-^CD4^-^CD8^-^ cell population expresses SC-1 (presumably Sca-1/Sca-2) antigen together with Thy-1; both appear on bone marrow-derived HSCs exposed to thymic hormones (49, 73, 81). This SC-1^+^PNA^+^ population of intrathymic TLPs exhibits high resistance to corticosteroids and γ-irradiation (49, 53, 68, 73), which likely is attributed to its efficient DNA damage repair mechanisms (140, 141). With modern knowledge, these TLPs may be considered as corresponding or closely related to CD117^+/-^CD44^+^CD25^-^ DN1 ETPs. In the adult mouse thymus, these DN1 ETPs demonstrate multipotent potential, giving rise at least to T, B, and dendritic cells, and presumably can be responsible for the early regeneration of the lymphoid compartment of the injured thymus, replacing radiosensitive bone marrow HSCs (140, 141).

To confirm that THGF acts specifically on early intrathymic TLPs, thymic cell suspensions were depleted of selected subpopulations before proliferation assays. Removal of SC-1^+^ TLPs or Thy-1^+^ cells completely abolished the proliferative response to THGF, whereas depletion of L3T4^+^ and Lyt-2^+^ mature thymocytes had no effect. Treatment of intact thymocytes with thymotropin, which downregulates SC-1 expression on early SC-1^+^Thy-1^+^ TLPs and promotes their maturation into SC-1^-^Thy-1^+^ cells (81, 82), also eliminated the THGF-dependent proliferative response (68, 104). Conversely, treatment of bone marrow cells with thymotropin converted them from the THGF-nonresponsive SC-1^-^ into THGF-responsive SC-1^+^ phenotype (68, 104) (**Table 6**), indicating a transition from SC-1^-^Thy-1^-^ HSCs to SC-1^+^Thy-1^+^ intrathymic TLPs (84).

Collectively, these results suggest that THGF targets a subpopulation of steroid-/radio-resistant early intrathymic progenitors SC-1^+^(Sca-1^+^/Sca-2^+^)PNA^+^Thy-1^+^ phenotype and stem cell properties, which are likely similar to CD117^+/-^CD44^+^CD25^-^ DN1 ETPs. However, dormant target cells of THGF probably do not express the CD117 receptor for SCF, or this expression is extremely low, at least up to their activation by THGF.

### 8.2 Peculiarities of THGF-induced prolonged proliferation of thymic cell cultures

The proliferative response to THGF was typically detected in 5-day thymocyte cultures, while the maximal response was observed on days 9-11. At this point, the number of viable thymocytes had decreased to 2–5% of the initial seeding density. During prolonged cultivation (up to 25–30 days), cell viability gradually increased but did not exceed 25-30% of the original input (51). Notably, the peak proliferative activity coincided with the minimal number of viable thymocytes. In contrast, the subsequent increase in cell numbers was associated with low spontaneous proliferation (51), suggesting complex integrative processes and the possible involvement of secondary messengers in THGF-induced thymic cultures.

To assess whether endogenous cytokines might influence this proliferative dynamics, either through THGF-induced secretion or as a result of thymocyte degradation, supernatants from thymocyte cultures, collected at various time points from cultures with or without THGF, were analyzed for THGF-, IL-2/4-, and IL-3-associated activities. Cross-reference bioassays employed cytokine-dependent cell lines and a standard thymocyte proliferation assay for THGF activity (51) (**Table 9**).

**Table 9:** Analysis of supernatants obtained from thymocytes pre-cultured with THGF or without THGF in CCM on the presence of THGF, co-mitogenic, IL-2, and IL-3 activities measured as a stimulation index in relation to spontaneous proliferation of corresponding test cultures.

Tested supernatants of pre-cultured mouse thymocytes:	Thymocytes^#^		CTLL-2^$^ IL-2/4-dependent	32D.c-1-23^$^ IL-3-dependent	STh-870^&**^ THGF-dependent
	Without Con-A	With Con-A			
Without THGF: 5 days	0.6 ± 0.1	9.9 ± 1.7	1.2 ± 0.1	0.6 ± 0.1	NT
10 days	0.6 ± 0.2	14.8 ± 1.5	1.2 ± 0.2	0.5 ± 0.1	NT
With THGF: 5 days	35.5 ± 3.5	58.2 ± 4.7	1.7 ± 0.2	1.2 ± 0.2	NT
10 days	60.0 ± 5.0	61.2 ± 4.5	2.5 ± 0.5	1.7 ± 0.2	NT
20 days	40.5 ± 3.8	56.9 ± 3.7	2.6 ± 0.4	3.4 ± 0.9	NT
10 days with THGF + 10 day without THGF	0.9 ± 0.1	6.0 ± 1.5	1.6 ± 0.2	1.5 ± 0.2	NT
24 h hours with THGF + 4 days without THGF	1.9 ± 0.3	1.1 ± 0.1	1.3 ± 0.2	1.1 ± 0.1	12.2 ± 1.8
With THGF: 75 days**	2.5 ± 0.3	1.4 ± 0.3	3.1 ± 0.5	1.7 ± 0.2	3.3 ± 0.5
97 days**	1.7 ± 0.2	3.4 ± 1.0	4.6 ± 0.9	1.2 ± 0.2	4.8 ± 1.0
Controls: IL-1	0.8 ± 0.2	12.9 ± 2.2	2.7 ± 0.3	1.1 ± 0.2	1.2 ± 0.1
IL-2	1.9 ± 0.3	15.3 ± 2.7	141 ± 10	3.9 ± 0.8	1.3 ± 0.2
IL-3	0.6 ± 0.1	3.0 ± 0.5	6.3 ± 1.0	45.0 ± 4.5	1.2 ± 0.2
THGF	21.2 ± 2.3	37.2 ± 2.7	5.9 ± 0.9	2.5 ± 0.3	11.8 ± 1.5
Spontaneous proliferation of test cultures in CCM (cpm)	199 ± 12	133 ± 8	219 ± 15	422 ± 27	60 ± 5

^#^5-day test cultures of intact CBA mouse thymocytes. ^$^24-hour proliferative test with IL-2- and IL-3-dependent cell lines (10^4^ cells/well, 4-hour [^3^H]thymidine incorporation). ^&^3-day proliferative test with 10^4^ cells/well and 20-hour [^3^H]thymidine incorporation. **THGF-dependent cell line STh-870 test cultures and supernatants on days 75, and 97 after growth initiation in the presence of THGF. CCM, complete culture medium; Con-A, concanavalin A; IL, interleukin; NT, not tested; THGF, thymocyte growth factor. Adopted from Shichkin, 1990 (51), Shichkin et al., 2015 (145). No permission required.

No THGF, IL-2/4, or IL-3 activity was detected in supernatants from 5- or 10-day thymocyte cultures maintained without exogenous THGF or other cytokines. However, these conditioned media demonstrated co-stimulating activity with Con-A, which may be associated with pre-accumulated IL-1, released from degrading thymocytes, or with other untested cytokines (51) (**Table 9**).

Remarkably, extended preculturing of thymocytes in the presence of exogenous THGF for 5, 10, and 20 days resulted in the appearance of presumably new THGF-like activity in the conditioned culture media, as well as co-stimulatory factors, likely corresponding to IL-1, IL-2/4, and possible IL-3, which appeared intermittently during THGF-dependent growth, especially for 10- and 20-day preculturing with exogenous THGF, probably as *de novo* synthesized factors. Analogous patterns were remarked for 75-day and 97-day thymocyte cultures supported in the constant presence of endogenous THGF supernatant (51) (**Table 9**).

Importantly, supernatants from thymocytes precultured with THGF for 24 hours and then maintained in fresh medium without THGF for an additional 4 days exhibited significant endogenous THGF activity in a highly sensitive proliferative assay using the THGF-dependent cell line STh-870, but no demonstrated IL-2 or IL-3 activities. These findings suggest the mitogenic role of THGF in initiating target-cell proliferation and supporting their subsequent autocrine-regulated growth, as well as possible induction of secondary growth and differentiation factors (51) (**Table 9**).

To further evaluate the contribution of other cytokines to THGF-induced proliferation, thymocytes cultured with exogenous THGF for 5, 10, 20, 97, and 126 days were tested for responsiveness to THGF, IL-1, IL-2, IL-3, and their combinations. Cytokine sources included J-774, EL-2, and WEHI-3 cell lines, respectively (51) (**Table 10**). Thymocytes preincubated with THGF exhibited elevated spontaneous proliferation and increased sensitivity to IL-1, IL-2, IL-3, and their combinations. In contrast, long-term cultures with low spontaneous proliferation (e.g., 97-day culture) required the presence of exogenous THGF to respond to these cytokines (51). These observations suggest that the thymic target cells for THGF are dormant TLPs, which, upon activation by THGF, acquire responsiveness also to other essential cytokines and become capable of further proliferation and differentiation within an appropriate intrathymic microenvironment.

**Table 10:** awThe proliferative response of long-lived and radioresistant thymic cell cultures to THGF and compared cytokines.

The proliferative response of CBA mouse thymocytes pre-cultured with THGF in CCM to THGF and interleukins, measured as a stimulation index in relation to spontaneous proliferation (cpm) of these thymocytes in CCM*												
Pre-culturation time (days)		CCM (cpm)	THGF	IL-1	IL-2	IL-3	THGF+IL-1	THGF+IL-2	THGF+IL-3	IL-1+IL-2	IL-1+IL-3	IL-2+IL-3
5		389	2.9	2.6	3.2	2.8	NT	5.1	NT	NT	NT	NT
10		734	4.8	1.8	6.8	4.3	9.4	9.3	9.1	5.5	7.7	9.9
20		410	5.1	3.2	3.6	4.4	9.0	11.7	7.6	2.4	3.0	7.0
97**		69	7.0	1.0	1.3	1.3	11.2	14.6	11.7	1.8	1.5	NT
126**		238	5.4	NT	6.8	6.3	NT	10.7	8.3	NT	NT	2.6

The proliferative response of intact thymocytes and irradiated with 15 Gy to THGF and interleukins after pre-culturation in CCM for 10 and 25 days, respectively, and to recombinant cytokines after irradiation with 50 Gy and pre-cultured for 25 days and 5 days without cytokines*												
Thymocyte cultures:					CCM (cpm)	THGF	IL-1	IL-2	IL-3	THGF + IL-1	THGF + IL-2	THGF + IL-3
Intact (10-day pre-culturation in CCM)					125	11.2	0.9	1.5	0.7	13.6	15.2	20.0
Irradiated 15 Gy (25-day pre-culturation in CCM)					120	26.6	1.1	1.7	0.5	28.5	43.3	23.3
Irradiated 50 Gy, pre-cultured in CCM and proliferative response:	IL-2	IL-4	IL-7	IL-9	SCF	GM-CSF	IL-2 + IL-4	IL-2 + IL-7	IL-2 + IL-9	IL-2 + SCF	IL-2 + GM-CSF	IL-7 + SCF
without PHA (25-day pre-culturation)	0.6	0.5	0.5	0.7	1.8	2.7	3.6	1.8	1.1	2.7	2.9	1.8
with PHA (25-day pre-culturation)	0.5	1.5	5.3	3.7	0.3	1.1	NT	NT	NT	NT	NT	NT
without PHA (5-day pre-culturation)	4.3	3.8	5.2	3.4	4.5	2.8	NT	NT	NT	NT	NT	NT

The proliferative response of irradiated thymocytes to THGF after preincubation in CCM for 5 days without growth factors*												
Proliferation type					Gamma irradiation doses (Gy)							
					10		20		30		50	
Spontaneous (cpm)					575 ± 62		90 ± 10		77 ± 8		75 ± 5	
THGF-induced (cpm)					3067 ± 210		2183 ± 180		1812 ± 160		1453 ± 123	
THGF-induced (SI)					5.3		24.2		23.5		19.3	
Response to IL-2 (SI)					2.0		1.8		1.5		1.3	

*3-day proliferative test with 10^4^ cells/well and 20-hour [^3^H]thymidine incorporation. **THGF-dependent cell line STh-870 on days 97, and 126 after growth initiation in the presence of THGF. CCM, complete culture medium; IL, interleukin; GM-CSF, granulocyte-macrophage colony-stimulating factor; SCF, stem cell factor; NT, not tested; SI, stimulation index; THGF, thymocyte growth factor. Adopted from Shichkin, 1990 (51), Shichkin et al., 2015 (145). No permission required.

Similar results were obtained in prolonged cultures of intact or irradiated (15 Gy) thymocytes grown without THGF or other growth factors for 10–25 days. These cultures exhibited only background levels of spontaneous proliferation, with cells remaining viable but quiescent throughout the observation period. At this, they retained strong responsiveness to THGF alone or in combination with IL-1, IL-2, or IL-3 (51) (**Table 10**).

Interestingly, high responsiveness to THGF, but not to IL-2, persisted across irradiation doses ranging from 10 to 50 Gy, although only 2-5% of thymocytes remained viable after irradiation. At this, spontaneous proliferation was minimal at 20–50 Gy, while irradiation at 10 Gy modestly enhanced proliferation, possibly through activation of endogenous cytokine secretion (51) (**Table 10**).

Furthermore, thymocytes isolated from sublethally irradiated mice and two days later irradiated *in vitro* with 50 Gy, and precultured for 5 or 25 days in cytokine-free medium, were then tested for proliferative responses to recombinant IL-2, IL-4, IL-7, IL-9, SCF, and GM-CSF, in the presence or absence of PHA. After 25 days of culture, irradiated thymocytes displayed minimal spontaneous proliferation and were unresponsive to IL-2, IL-4, IL-7, IL-9, and SCF, showing only a weak response to GM-CSF in the absence of PHA. The addition of PHA induced co-stimulation primarily with IL-7 and IL-9, and modestly enhanced reaction to combinations of IL-2 with IL-4, SCF, or GM-CSF. In contrast, 5-day cultures of irradiated thymocytes responded to these cytokines in the direct proliferative test (7) (**Table 10**).

Collectively, these data support the assumption that long-lived, irradiated thymocytes maintain proliferation through autocrine mechanisms provided by THGF. The tested cytokines may also contribute to their survival and proliferation, presumably following the primary activation by THGF. These findings also suggest that the thymic cells surviving irradiation represent radioresistant TLPs, which remain sensitive to growth factors typical for HSCs and TLPs. Given that THGF activates these cells at concentrations over 3,000-fold lower than other cytokines, while inducing a markedly stronger proliferative response, THGF appears to function as the primary activating signal among these cytokines. However, their efficient proliferation and differentiation also require the presence of other cytokines of the intrathymic network.

### 8.3 Impact of IL-2 and γ-irradiation on THGF-responding cells

Some population of CD4^-^CD8^-^ TLPs respond to IL-2 only after activation by mitogens (85) or in the presence of IL-1 (142, 143). THGF-responsive cells do not initially respond to IL-2; however, pre-incubation with THGF induces their sensitivity to IL-2. Moreover, THGF and IL-2 act synergistically to enhance thymocyte proliferation (51) (**Table 10**).

THGF-responsive thymocytes display notable resistance to both sublethal γ-irradiation *in vivo* and to doses up to 50 Gy *in vitro*. Neither hydrocortisone treatment nor γ-irradiation (10–50 Gy) affected the responsiveness of thymocytes to THGF during long-term culture in the absence of exogenous cytokines, including THGF itself (51, 53) (**Table 10**). Pre-incubation of viable irradiated thymocytes or freshly isolated thymic cells with THGF significantly enhanced their IL-2 responsiveness. In contrast, pre-incubation with IL-2 led to the accumulation of IL-2-responsive cells that were unresponsive to THGF (51, 72).

These findings suggest that THGF-responsive TLPs are initially negative for expression of IL-2R or express its non-active low-affinity form, and exposure to THGF induces the expression of high-affinity IL-2 receptors, similar to the activating effect of IL-1 or mitogens on thymocytes sensitive to IL-2. Importantly, THGF probably does not use IL-2R for its signaling, as blocking the IL-2Rα chain (CD25) with specific monoclonal antibodies abolished IL-2-dependent proliferation but did not affect THGF-induced proliferation of thymocytes (75, 76) (**Table 7**).

As is known, the IL-2 receptor system comprises three subunits: low-affinity IL-2Rα (CD25), IL-2Rβ (CD122), and the common γ chain IL-2Rγ (CD132). At this, only IL-2Rα can bind IL-2 with low affinity, which does not lead to cell activation. The β/γ heterodimer forms an intermediate-affinity receptor, whereas the α/β/γ trimeric complex confers high-affinity binding and full-fledged functional signaling (144). However, the intermediate receptor form is also capable of transducing IL-2-dependent signals (144). This suggests that IL-2 and other cytokines utilizing the IL-2Rγ chain, such as IL-4, IL-7, IL-9, IL-13, IL-15, and IL-21 (144), may serve as secondary or co-stimulatory signals that cooperate with THGF during thymocyte activation and prolonged proliferation.

In contrast, THGF itself likely utilizes a distinct receptor system, presumably the Sca-1/Sca-2 complex or another, yet unidentified receptor system, to mediate primary activation of its target cells. Thus, non-activated cortisol-/radioresistant SC-1^+^(Sca-1^+^/Sca-2^+^)PNA^+^Thy-1^+^CD25^-^CD4^-^CD8^-^ intrathymic TLPs, represent the principal target population for THGF. THGF stimulates these TLPs to express the high-affinity IL-2R complex, thereby transitioning them from a THGF-sensitive to a THGF/IL-2-sensitive stage.

Collectively, these data provide evidence for the specificity of THGF-dependent thymocyte proliferation and establish a sequential model of TLP activation from initial THGF-mediated priming to IL-2/IL-2R-dependent expansion. Consequently, THGF likely represents the key trigger factor in the post-irradiation regeneration of the lymphoid thymic compartment, bridging the activation of radioresistant progenitors with their further proliferation driven by IL-2 and other cytokines (52).

### 8.4 Clonally cluster-forming growth and morphology of radioresistant and THGF-dependent long-lived thymic cell cultures

**Figure 2:**
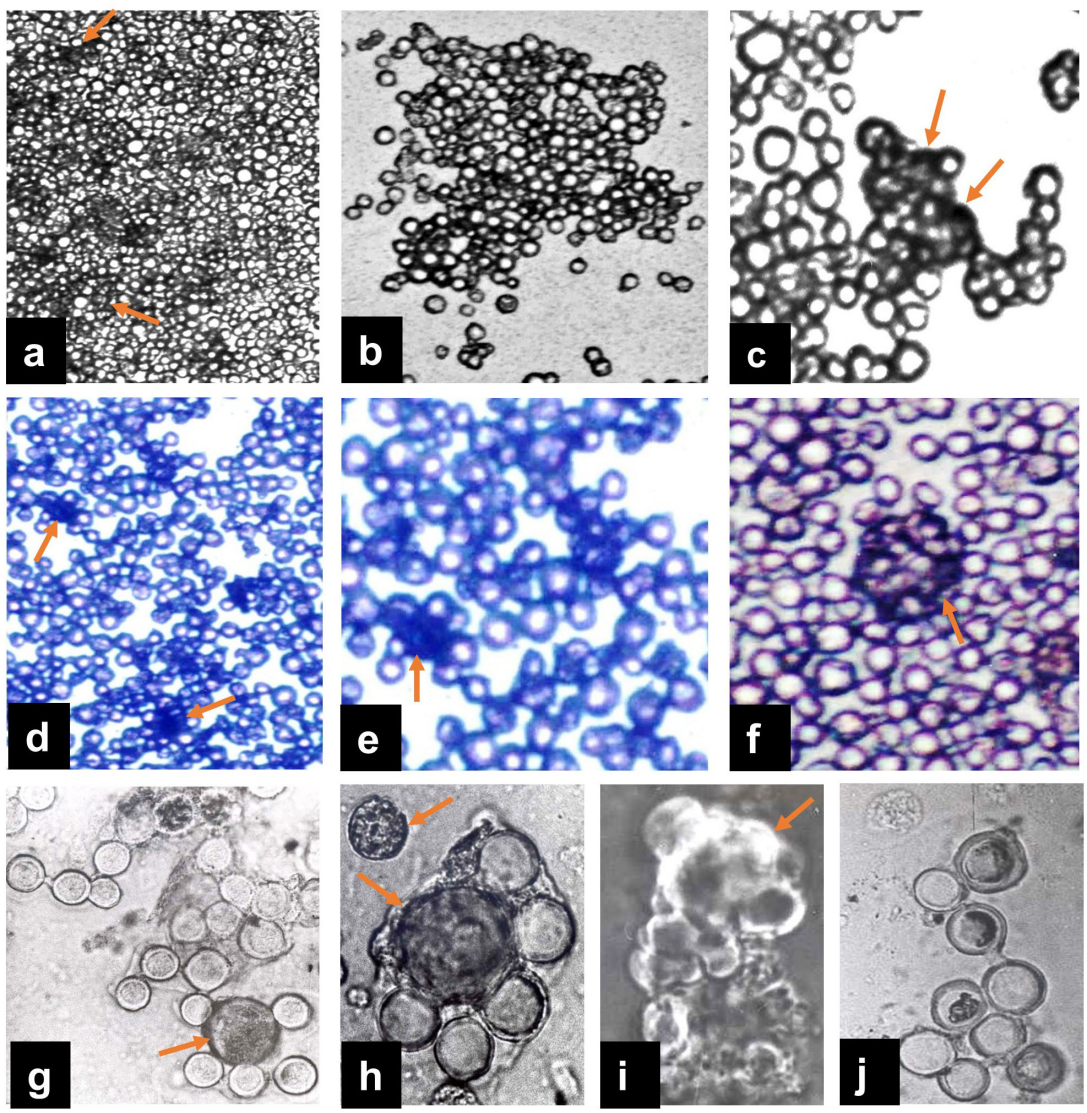
Clonally-cluster growth and morphology of radioresistant and THGF-dependent long-lived thymic cell cultures. **(a – f)**, 90-day culture of irradiated with 50 Gy thymocytes prepared from sublethally irradiated (4 Gy) CBA mice. **(a–c)**, non-stained; **(d–f)**, stained with eosin and hematoxylin. Light microscopy, x200 magnification. **(g–j)**, THGF-dependent thymic cell line STh-870 in the growth period from 320 to 410 days after initiation. Light microscopy, x700 magnification. Arrows show the clone-/cluster-forming mother cells, presumably activated T-lymphocyte precursors (TLPs) in the stage of double-negative early TLPs (DN1 ETPs), which are surrounded by smaller daughter cells, presumably in DN2→DN4 TLP transition stages. Adopted from Shichkin, 1990 (51), Shichkin and Durum, 2000 (124), Shichkin et al., 2015 (145). No permission required.

Radioresistant thymic cells isolated from CBA mice 2 days after irradiation of mice with 4 Gy, and *in vitro* with 50 Gy, which survived in prolonged culture using regular culture medium without supporting growth factors, formed visible clones by day 30. By this time, the proportion of viable thymocytes progressively declined to 10–15%, whereas by day 90, cell viability had increased to nearly 95%, and the clones had reached substantial size. The 90-day clones were fixed and stained directly in the same culture chambers in which the irradiated cells had initially been seeded (124, 145). Although this long-term culture was maintained without exogenous THGF or other cytokines, the formation of characteristic rosette/cluster structures, comprising single “mother” cells (large, dark cells) surrounded by “daughter” cells, were observed. These structures were morphologically similar to those previously described for the long-lived THGF-dependent thymocyte line STh-870 (51) (**Figure 2**).

Considering that radioresistant thymic cells in our experiments were unable to proliferate in response to various cytokines without prior incubation with THGF, and given that the combined two-phase irradiation (mice *in vivo* followed by thymocytes *in vitro*) induced pronounced THGF-like activity in thymocyte cultures harvested 2 days after *in vivo* irradiation, as well as the sustained survival and vigorous long-term proliferation leading to the formation of large clones/clusters, suggest expression of THGF-like activity in these cultures, functionally substituting for exogenous THGF.

The morphological and proliferative features of the THGF-dependent STh-870 line, documented by hourly imaging from day 320 to day 410, allowed the distinction of at least three sequential growth stages (51) (**Figure 2**). The first stage: a resting stage (G0), is represented by single large dark cells, which correspond presumptively to DN1-type TLPs (dormant ETPs). The second stage: activation and synthesis (G1–S) in the absence of visible new cells, is tentatively also associated with DN1 (activated ETPs). During this stage, single “mother” cells, as assumed, are capable of “defended” mitosis and/or amitotic division, and active DNA synthesis due to a mechanism of “daughter” cell formation through this defended mitosis inside mother cell-like structures. The third stage: cluster formation, likely corresponds to DN2–DN4 transitions. Daughter cells emerge on the surface of activated mother cells, initially forming rosette structures of 5–10 cells, and then larger clusters. Fully formed clones/clusters of daughter cells typically contain 30–50 cells and are observed both in the THGF-dependent cell line STh-870 and in the long-lived clones formed by irradiated thymocytes (**Figure 2**).

Notably, long-term maintenance of the STh-870 line required periodic replacement (every 10–15 days) of THGF-containing medium, with each fresh THGF addition triggering a new cycle of cluster formation. The entire cycle, from activation of a resting cell to the formation and subsequent death of the cluster, spanned 20–30 days, closely resembling the early phase of thymus regeneration *in vivo* in mice following sublethal γ-irradiation (51, 55, 107). In contrast, the long-lived irradiated thymic cell cultures did not exhibit comparable cluster-formation cyclicity. Instead, their continuous clonal expansion and sustained high viability indicate the persistent presence of THGF-like activity, resulting in the simultaneous development of multiple clones, as observed in the described experiment.

### 8.5 Impact of colchicine on THGF-dependent proliferation of thymocytes

To clarify the causes of the atypical dynamics of thymocyte proliferation and viability in the presence of THGF, as well as unusual morphological structures of long-lived radioresistant and THGF-dependent thymocyte cultures, we examined the effect of colchicine, an inhibitor of mitosis, on THGF-dependent proliferation in a 5-day test culture, compared to Con A- and IL-2-induced proliferation. We found that colchicine at an active concentration of 2.5 μg/ml did not inhibit THGF-dependent thymocyte proliferation, whereas it completely blocked Con A- and IL-2-induced proliferation, as assessed by [³H]thymidine incorporation (53) (**Table 11**).

**Table 11:** Effect of colchicine on THGF-, Con A-, and IL-2-dependent thymocyte proliferation and phenotypic characteristics of long-lived radioresistant thymic cell cultures.

Test type	Test components		Concentration (%)		Proliferative level	
					cpm	Stimulation index
5-day test	THGF		50.0		3887 ± 522	15.7
			25.0		3296 ± 513	15.3
			12.5		3448 ± 714	13.9
			0.0		247 ± 39	1.0
	THGF (%) + Colchicine (2.5 μg/ml) Colchicine (2.5 μg/ml)		50.0 25.0 12.5 0.0		2629 ± 377 3271 ± 213 2112 ± 767 210 ± 30	12.5 15.5 10.0 1.0
3-day test	Con A (μg/ml)		10.0		8517 ± 303	9.8
			5.0		24792 ± 1544	69.6
			2.5		1924 ± 84	5.4
			0.0		356 ± 52	1.0
	Con A (μg/ml) + Colchicine (2.5 μg/ml) Colchicine (2.5 μg/ml)		10.0 5.0 2.5 0.0		475 ± 188 274 ± 25 333 ± 14 350 ± 47	1.3 0.7 0.9 1.0
3-day test	IL-2 (%) + Con A (2.5 μg/ml)		50.0 25.0 12.5 0.0		21325 ± 1923 9512 ± 895 1145 ± 226 170 ± 97	125.4 55.9 6.5 1.0
	IL-2 (%) + Con A (2.5 μg/ml) + Colchicine (2.5 μg/ml) Colchicine (2.5 μg/ml)		50.0 25.0 12.5 0.0		348 ± 96 287 ± 120 203 ± 98 155 ± 17	2.2 1.8 1.3 1.0

Phenotype	Percent of thymocytes γ-irradiated *in vivo* with 4 Gy and *in vitro* with 50 Gy and cultured for:				Non-irradiated thymocytes (%)	
	5-day	10-day	25-day	90-day	5-day	10-day
CD4^-^CD8^-^	4	1	27	76	4	0.5
CD4^+^CD8^+^	79	86	31	18	80	80
CD4^+^CD8^-^	11	10	19	3	10	18
CD8^+^CD4^-^	6	3	23	3	6	1.5
CD25^-^CD44^-^	NT	25	NT	74	NT	42
CD25^+^CD44^+^	NT	9	NT	22	NT	7
CD25^+^CD44^-^	NT	64	NT	3	NT	50
CD44^+^CD25^-^	NT	2	NT	3	NT	1

Con A, concanavalin A; NT, not tested. Adopted from Shichkin, 1992 (53), Shichkin and Durum, 2000 (124), Shichkin et al., 2015 (145). No permission required.

Colchicine is known to arrest the cell cycle at the G2/M transition and exert significant cytotoxicity by inducing oxidative stress, decreasing mitochondrial membrane potential, increasing DNA damage, and triggering apoptosis. By disrupting spindle formation during metaphase, colchicine prevents normal mitotic progression, causing cells to revert to a prometaphase-like state (146).

Thus, the observation that colchicine fails to inhibit THGF-induced proliferation is highly unexpected and difficult to reconcile with classical cell biology. This finding suggests at least two possible futures:

(1) The plasma membrane of THGF-responsive cells may be impermeable to colchicine at least during the early phase of THGF exposure. Therefore, THGF-induced DNA synthesis may proceed as “defended mitosis” inside mother cell-like structures, as described above.

(2) Such colchicine-insensitive proliferative behavior is also consistent with processes such as amitotic or endomitotic cell division, which may underlie the early stages of THGF-driven proliferation.

In other words, THGF appears to trigger DNA synthesis in responsive cells before the emergence of daughter cells, which, as assumed, may form internally within the mother cell using this new DNA material. This could explain the characteristic proliferative peak at days 5–10, followed by the subsequent appearance of cell clones or clusters and an increase in total cell number in the prolonged cultures.

### 8.6 Phenotypic profile of long-lived radioresistant thymic cell cultures

Phenotypic profile of prolonged thymic cultures generated by thymocytes isolated from sublethally irradiated CBA mice and irradiated additionally *in vitro* with a dose of 50 Gy, was analyzed on day 5, 10, 25, and 90 of continuous growth in the same culture chambers with periodic change of cytokine-free medium (**Table 11**).

By day 10, cultures of the irradiated thymocyte were composed predominantly of CD4^+^CD8^+^ DP immature thymocytes and CD25^+^CD44^-^ cells, presumably related to DN3 TLPs. Minor populations included CD4^-^CD8^-^, CD4^+^CD8^-^, and CD8^+^CD4^-^ cells, as well as CD25^-^CD44^-^ cells (presumably DN4 TLPs), CD44^+^CD25^-^ (presumably DN1 TLPs), and CD44^+^CD25^+^ (presumably DN2 TLPs) (**Table 11**).

In contrast, by day 90, the majority of cells (74-76%) exhibited a CD4^-^CD8^-^CD25^-^CD44^-^ phenotype characteristic of DN4 TLPs. Immature DP CD4^+^CD8^+^ thymocytes consisted 18%, and mature SP CD4^+^CD8^-^ and CD8^+^CD4^-^ thymocytes were each only 3%. CD44^+^CD25^+^ TLPs (DN2) were presented by 22%, while CD44^+^CD25^-^ (DN1) and CD25^+^CD44^-^ (DN3) TLPs were also presented as minor populations (3%) (**Table 11**).

Collectively, these findings suggest that the dominant population in 90-day cultures of irradiated thymocytes consists of CD4^-^CD8^-^CD25^-^CD44^-^ TLPs. This phenotype corresponds to the latest stage of TLPs maturation (DN4), and these cells presumably correspond to the daughter cells that form THGF-induced clusters both in the THGF-dependent STh-870 cell line and in irradiated long-lived thymocyte cultures (51, 124) (**Figure 2**). The large “mother” cells observed in these cultures are presumably dormant DN1-stage cells, which, upon activation, transfer to DN2 stage and give rise to forming rosette and cluster structures mainly formed by DN4 TLPs as a result of the irradiation-induced arrest of further differentiation at the DN3-DN4 stage, associated with TCRαβ formation and dependent on Notch-signaling (147–149).

## 9 Incited facts and hypotheses

**Figure 3:**
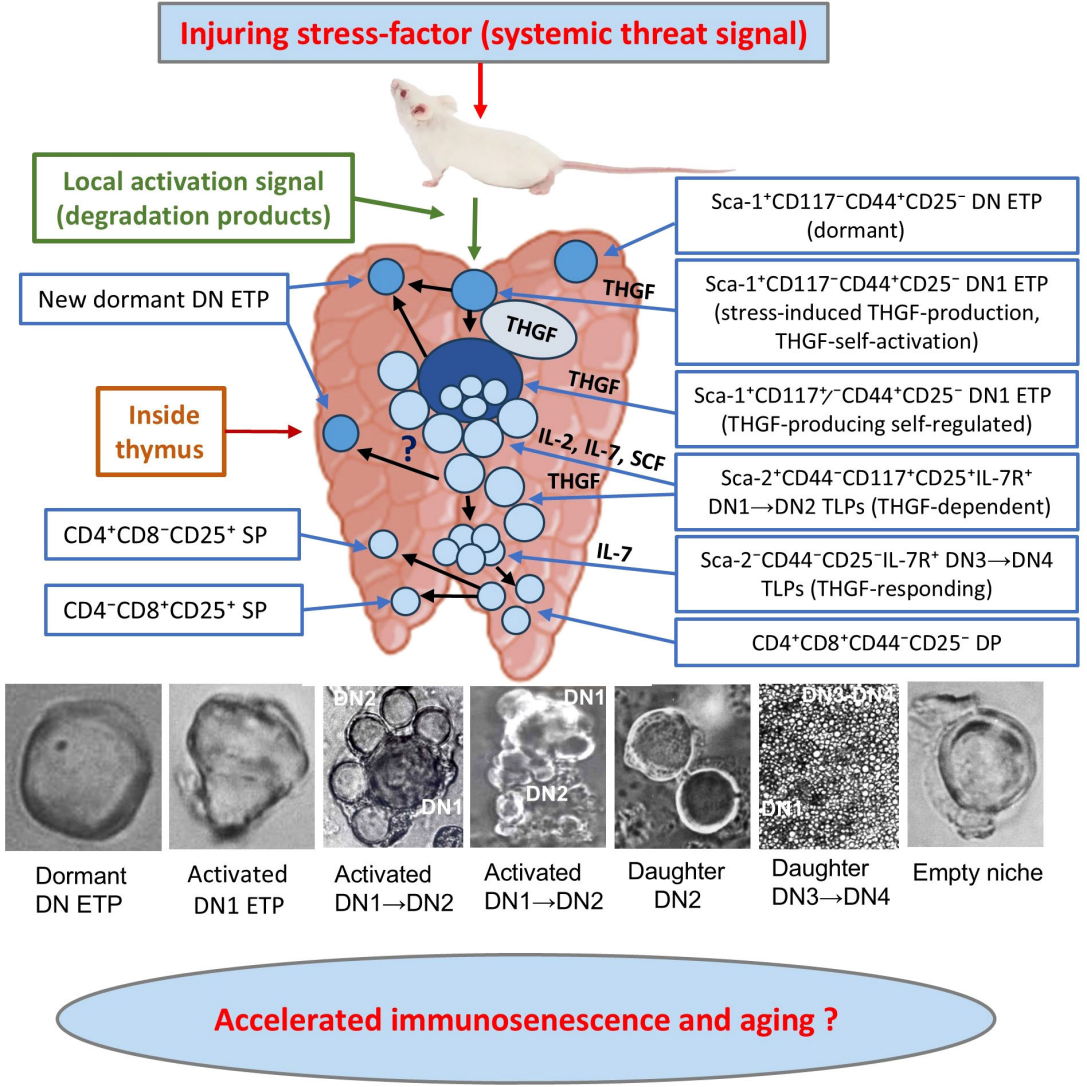
Hypothetical model of THGF-dependent pathway activation of radioresistant intrathymic stem cells and role in lymphoid compartment recovery after thymic damage. The diagram illustrates the hypothesized sequence of cellular events following irradiation-induced systemic stress, leading to the activation of dormant DN CD4^-^CD8^-^ early thymocyte precursors (DN1 ETP stage) and the synthesis of THGF. Upon autocrine self-stimulation by THGF, these cells are assumed to initiate a protected intracellular formation of daughter cells, primarily at the DN1 ETP stage, bypassing conventional mitosis. The process is characterized by high radioresistance, insensitivity to colchicine, and potential for multipotent differentiation, and is presumably completely THGF-dependent. The subsequent maturation of THGF-activated intrathymic TLPs through DN2-DN4 TLP stages up to DP CD4^+^CD8^+^ thymocytes suggests cooperation with IL-22-dependent radioresistant stromal-epithelial microenvironment and involvement of other critical cytokines (SCF, IL-7, IL-2). The model illustrates the concept of THGF as a key autocrine factor in emergency activation of reserve intrathymic stem cells and their self-renewal under conditions of systemic damage, which, when repeatedly retriggered, may lead to the depletion of dormant stem cells, accelerated immunosenescence, and premature aging due to restricted regeneration of lymphoid pools, therefore addition the concept of IL-22-dependent regeneration of thymic stromal-epithelial tissue. It is assumed that the THGF-dependent pathway of thymopoiesis is activated only under conditions of damaging effects and is not involved in conditions of normal thymopoiesis. DN, double-negative; DP, double-positive; ETP, early thymocyte precursor; IL, interleukin; SCF, stem cell factor; SP, single-positive; TLP, T-lymphocyte precursor; THGF, thymocyte growth factor. Photography of cells adopted from Shichkin, 1990 (51), Shichkin et al., 2015 (145). No permission required.

Retrospective analysis of our early published data, summarized in this review, generally supports our conclusions regarding the nature and novelty of THGF and the unique properties of its target cells, presumably dormant radioresistant intrathymic stem cells. This analysis also clarifies the most probable position of THGF-producing and THGF-responding TLPs within the modern intrathymic hierarchy, placing them at the stage of non-activated DN ETPs with the integrated phenotype CD117^-^Thy-1^+^Sca-1^+^CD44^+^CD25^-^CD4^-^CD8^-^, which, under THGF activation, express functional IL-2R and, presumably, c-kit receptor for SCF, transferring to DN1-DN2 stages (**Figure 3**).

The properties of the THGF-dependent long-term STh-870 cell line suggest that THGF induces not only robust DNA synthesis but also intracellular formation of new daughter cells. These processes occur predominantly within the first 15 days after dormant stem cells receive the THGF signal. During this period, the most intense wave of DNA synthesis is observed, yet without an increase in cell number. And what is critically important, this synthesis is fully insensitive to colchicine. At the end of this phase, only single blast-like cells remain visible in culture; however, over the subsequent 10–15 days, they give rise to rosettes and, later, clusters of 30–50 daughter cells. This second phase is accompanied by a moderate increase in DNA synthesis and a rise in viable cell numbers.

The intracellular generation of new THGF-dependent cells may be advantageous under extreme conditions, such as radiation injury, where classical mitosis is impaired. Morphological comparisons, coupled with the colchicine-resistant proliferative response, suggest that large “mother” cells serve as primary recipients of THGF signals, within which daughter cells are formed. In this context, the mother cell functions as a germinal-like center or a spheroid niche, enabling the protected formation of new cells. Such a mechanism could account for the absence of colchicine effects on THGF-driven proliferation, implying that dormant stem cells employ this strategy for rapid, protected expansion before further differentiation.

The proposed mechanism of intracellular or intra-spheroid generation of new daughter cells may involve a combination of defended mitosis or amitotic division in mother cells and defended mitosis or endomitotic processes in daughter cells. This hypothesis helps explain both the colchicine insensitivity and the pronounced radio resistance of THGF-responding cells, as amitotic and endomitotic division are inherently less vulnerable to radiation-induced damage. Such mechanisms may have evolved as ancient anti-radiation strategies preserved in primitive dormant stem cell populations.

Taken together, these observations suggest that mother cells in long-lived THGF-dependent cultures, as well as analogous radioresistant cells, are likely dormant self-renewing intrathymic CD4^-^CD8^-^ multipotent stem cells at the DN1 ETP stage, which can be activated either by THGF or irradiation. Their progeny, the smaller cells, correspond to the DN2-DN4 transition stages. After activation by THGF, these TLPs may acquire responsiveness to SCF, IL-2, and IL-7 and potentially may generate immature DP CD4^+^CD8^+^ and SP CD4^+^ and CD8^+^ thymocytes. However, their continued differentiation requires additional cytokines (IL-2, IL-4, IL-7) and interaction with stromal–epithelial microenvironment that are absent *in vitro*, eventually leading to the cluster degradation (**Figure 3**).

Although the concept of intracellular formation of daughter-cell pools may seem unrealistic, it is supported by several analogous observations. Thymic epithelial nurse cells are known to internalize immature CD4^+^CD8^+^ thymocytes and provide intracellular sites for TCRγδ cell maturation (150, 151). Similar rosette-like structures include thymic rosettes formed by thymocytes associated with intrathymic macrophages or dendritic cells (152, 153). While we did not find these cell types in THGF-responding long-term cultures, their possible involvement in THGF-dependent growth and cluster formation should also be brought to attention. Thus, verification of the single cells in the clusters remains a subject of further studies and debate, as well as the nature of “mother cells”.

The proliferative response of the long-lived THGF-dependent STh-870 line to THGF, interleukins, and mitogens was fundamentally similar to those of freshly isolated thymocytes, irradiated long-lived thymocytes, or thymocytes pre-incubated with THGF. However, this response was time-dependent after the addition of a fresh THGF portion, and the level of spontaneous proliferation, which supports the conclusion that THGF induces the expression of high-affinity receptors for other cytokines, and the activation of the THGF signaling pathway is a priority in the chain of further events.

Our experimental results show that the target cells of THGF belong to a radioresistant intrathymic stem-cell population with a D_0_ exceeding 50 Gy. Irradiation presumably induces the activation of dormant radioresistant DN1 TLPs and secretion of endogenous THGF by these cells, which then regulates their proliferation via an autocrine loop and induces their transition to the CD25^+^ DN2 stage, further controlled by IL-2 and IL-7. This interpretation is consistent with evidence that radioresistant DN2 TLPs proliferate after irradiation in an IL-7-dependent manner, generating conventional thymocytes and using the IL-2/IL-2R pathway during progression (52, 85, 140).

As is known, some DN1-DN2 TLPs possess multipotent potential and can generate not only T cells but also NK cells (154), dendritic cells (155), macrophages, and B cells (156). These cells may secrete IL-7, SCF, and other cytokines that support thymic regeneration *in vivo* after irradiation. These findings also assume the possibility that THGF-dependent, or macrophage- or dendritic cell-associated rosette-like structures could arise through self-organization from single multipotent TLPs due to their plasticity and multipotency.

A recently described subpopulation of radioresistant TECs (58, 59, 62) may also contribute to the postirradiation restoration of the thymic function by producing essential cytokines and providing signaling pathways for intercommunication with radioresistant TLPs (57, 60, 61), as well as producing chemokines such as CCL19, CCL21, and CCL25, which are important for the attraction of TLPs (20, 122).

Furthermore, innate lymphoid cells 3 (ILC3) and T helper 17 (Th17) cells produce a cytokine (IL-22) that is crucial for the thymic epithelial compartment recovery after high-dose chemotherapy or irradiation damage (136–138). Defects in IL-22 production delay thymus recovery in irradiated mice and decrease the expression of genes Foxn1, Aire, and Kgf, associated with thymic function. In contrast, the administration of IL-22 facilitates the repair of TECs, increases the number of T cells, increases the level of Aire, and increases the proportion of natural regulatory T cells in the thymus (139).

Notably, following total body irradiation or targeted thymus irradiation, which leads to crucial depletion of DP thymocytes, the level of intrathymic IL-22 has been increased, suggesting a link between IL-22 and mechanisms of endogenous recovery (136, 138, 157) similar to the effect of THGF on TLPs. Furthermore, production of IL-22 following damage is related to radioresistant innate thymic LTi/ILC3 cells, whose number increases following thymic insult (20, 136). This is also similar to the THGF effect, increasing SC-1^+^ TLPs in the thymus upon injection into mice (104).

These data suggest the need for further evaluation of THGF-responding/producing cells in comparison with the LTi/ILC3 population, as well as additional identification of THGF, THGF-specific receptors, and key molecular regulators of the THGF-signaling transduction pathways.

## 10 Concluding remarks

The collective evidence presented in this review supports the concept that THGF is a key regulator of early intrathymic stem cells, playing an initiating role in the regeneration of the lymphoid compartment under stress conditions.

THGF-producing and THGF-responding cells are likely localized at the earliest DN1/DN2 stages of thymocyte development and display properties characteristic of primitive, radioresistant intrathymic stem cells.

Their unique response to THGF distinguishes THGF-driven proliferation from classical cytokine-induced mitosis.

THGF presumably initiates the earliest step in a hierarchical sequence of cytokine responsiveness, enabling subsequent sensitivity to IL-7, SCF, IL-2, and other mediators of thymocyte expansion and differentiation.

The ability of γ-irradiation to trigger THGF secretion further highlights its physiological relevance in thymic repair.

Insights into THGF-dependent mechanisms of radio resistance in parallel with recent discoveries concerning radioresistant TEC subsets and LTi/ILC3 populations that contribute to post-irradiation thymic recovery, suggesting that THGF may operate within a broader reparative network.

The proposed models of daughter cell generation in the conditions of defended mitosis or amitotic/endomitotic cell division provide a reasonable explanation for both the exceptional radio resistance and the morpho-proliferative peculiarities of THGF-sensitive TLPs, as well as unresponsiveness to colchicine effect, potentially representing an evolutionarily conserved mechanism for maintaining thymopoiesis and possible hematopoiesis under extreme conditions.

The properties of THGF to stimulate the development of colonies/clusters in long-lived THGF-dependent thymic cultures, as well as colony-formation in spleen, suggest its similarity to other colony-stimulating factors, such as SCF, GM-CSF, and IL-3. However, a range of parameters suggests an independent role of THGF extended beyond the thymus and requires further verification.

Further molecular identification of THGF and its receptors in long-lived thymic cell cultures, induced by irradiation and THGF-associated signaling pathways, as well as of these radioresistant cells nature and interaction of THGF with other cytokine pathways, may provide essential information for verification of THGF biology and understanding of thymic homeostasis in the context of the thymus recovery after irradiation and other injuring actions, providing new potential for developing therapeutic approaches to immune system reconstitution.

## Data Availability

The original contributions presented in the study are included in the article/supplementary material. Further inquiries can be directed to the corresponding author.
